# Targeting PTBP3‐Mediated Alternative Splicing of COX11 Induces Cuproptosis for Inhibiting Gastric Cancer Peritoneal Metastasis

**DOI:** 10.1002/advs.202415983

**Published:** 2025-04-24

**Authors:** Yajing Zhou, Chao Dong, Xiaochun Shen, Pengbo Wang, Tao Chen, Weikang Li, Xiaotong Sun, Peiyuan Li, Chengxiang Xu, Kaipeng Duan, Dongbao Li, Jin Zhou

**Affiliations:** ^1^ Department of General Surgery The First Affiliated Hospital of Soochow University 899 Pinghai Road Suzhou Jiangsu Province 215031 China; ^2^ Department of Pulmonary and Critical Care Medicine The First Affiliated Hospital of Soochow University 899 Pinghai Road Suzhou Jiangsu Province 215031 China

**Keywords:** alternative splicing, antisense oligonucleotide drugs, cuproptosis, gastric cancer peritoneal metastasis, patient‐derived organoids‐based xenograft

## Abstract

Numerous aberrant splicing events are implicated in tumor progression, yet comprehensive reports on splicing factors and events associated with peritoneal metastasis in gastric cancer (GCPM) are lacking. In this study, PTBP3 is found to be significantly overexpressed in peritoneal metastatic tissues of gastric cancer compared to primary tumor tissues, and higher PTBP3 expression correlates with poorer prognosis. Using gastric cancer cells and patient‐derived organoids (PDO), the role of PTBP3 in promoting tumor invasion and proliferation is investigated. Mechanistically, through full‐length transcriptome sequencing, PTBP3 mediates exon 4 skipping in its target gene COX11, leading to shorter transcripts that impair COX11 protein function, reducing mitochondrial copper content and enabling tumor cells to evade cuproptosis. Antisense oligonucleotide (ASO) drugs targeting the short COX11 transcripts effectively degrade mRNA, disrupting copper homeostasis. In PDO‐based xenograft models, exogenous copper ionophores combined with ASO drugs induce excessive copper accumulation in mitochondria, triggering proteotoxic stress and cuproptosis. Overall, PTBP3‐mediated exon 4 skipping in COX11 pre‐mRNA is critical for tumor cell survival and progression in GCPM, offering potential therapeutic strategies targeting copper metabolism.

## Graphical abstract

The splicing factor PTBP3 promotes COX11 exon skipping, allowing gastric cancer organoids to evade cuproptosis. Antisense oligonucleotide drugs targeting PTBP3‐mediated COX11 alternative splicing, in combination with copper ionophores, promote cuproptosis in organoids, thereby providing a therapeutic approach for gastric cancer peritoneal metastasis.

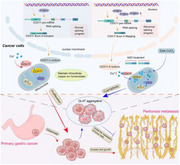

## 1 Introduction

Gastric cancer is one of the most prevalent malignant tumors of the digestive system worldwide. According to the latest national cancer statistics released in 2022, gastric cancer ranks third in China for both new cases and deaths, significantly surpassing those of other countries.^[^ ^1^ ^]^ The peritoneum is the most common site of recurrence and metastasis in advanced gastric cancer, with a five‐year survival rate of less than 2% for patients presenting with peritoneal metastasis.^[^ ^2^, ^3^ ^]^ The “seed and soil” theory is one of the most widely accepted explanations; however, the mechanisms underlying peritoneal metastasis remain incompletely understood.^[^ ^4^ ^]^ Diagnosis is typically confirmed through imaging studies, serum biomarker detection, laparoscopic examination, and the identification of free cancer cells in the peritoneal cavity. Common treatment modalities include surgical intervention, postoperative adjuvant chemotherapy, and intraperitoneal hyperthermic chemotherapy.^[^ ^5^ ^]^ Gastric cancer exhibits significant heterogeneity, leading to an increasing emphasis on the precision of immunotherapy approaches for this malignancy.^[^ ^6^ ^]^ Multi‐omics approaches are facilitating the development of more accurate subtyping of gastric cancer, with the aim of identifying additional therapeutic targets.^[^ ^7^ ^]^

During pre‐mRNA processing, introns are excised, and exons are spliced together through alternative splicing patterns. The sequential joining of exons is termed constitutive splicing, while processes such as exon skipping, intron retention, and the use of cassette exons are classified as alternative splicing.^[^ ^8^ ^]^ Many critical genes undergo alternative splicing in tumors, influencing diverse phenotypes associated with tumor initiation and progression.^[^ ^9^, ^10^, ^11^, ^12^ ^]^ Cryo‐electron microscopy has provided high‐resolution images of the spliceosome, offering critical insights into the structural dynamics of RNA–protein complexes in structural biology.^[^ ^13^, ^14^, ^15^ ^]^ In this context, splicing factors, as integral components of the spliceosome, play a crucial role.^[^ ^16^ ^]^ Therefore, we further investigate the regulatory processes and expression changes of splicing factors in the context of gastric cancer peritoneal metastasis (GCPM). This focus could enhance our understanding of their specific roles in cancer development and provide new therapeutic targets.^[^ ^17^ ^]^ The relationship between splicing factors and tumor metastasis has been extensively studied.^[^ ^18^, ^19^ ^]^ Building on these findings, further investigation is needed to elucidate the role of splicing factors in the mechanisms driving peritoneal metastasis of gastric cancer.

PTBP3, a member of the polypyrimidine tract‐binding protein (PTBP) family, plays a crucial role in RNA splicing, translation activation, and mRNA stabilization. Numerous studies have demonstrated that PTBP3 functions as an oncogene, promoting tumor progression.^[^ ^20^ ^]^ However, the role of PTBP3 and its mechanisms in GCPM remain incompletely understood.

Cuproptosis is a recently discovered mechanism of cell death distinct from previously recognized mechanisms.^[^ ^21^ ^]^ Cuproptosis occurs when copper ions bind directly to lipoylated components of the tricarboxylic acid cycle within mitochondria. This interaction leads to the aggregation of lipoylated protein oligomers and the downregulation of iron–sulfur cluster proteins, resulting in protein toxicity and ultimately cell death. Numerous studies have demonstrated that copper plays a significant role in various processes associated with tumorigenesis.^[^ ^22^ ^]^ However, research on cuproptosis in gastric cancer is limited, and the mechanisms underlying its involvement in gastric cancer metastasis remain largely unexplored.^[^ ^23^ ^]^ Exploiting cuproptosis mechanisms may provide new therapeutic strategies for gastric cancer treatment. Therefore, further investigation into the relationship between cuproptosis and tumor metastasis is needed.

In this study, we analyzed single‐cell data and found that PTBP3 is overexpressed in GCPM tumor cells, correlating with poor patient outcomes. Full‐length transcriptome sequencing revealed that PTBP3 promotes exon 4 skipping in COX11, generating two transcript isoforms. Notably, the shorter isoform is significantly upregulated in metastatic tissues. We developed an antisense oligonucleotide (ASO) drug targeting the short COX11 transcript, which increases intracellular copper levels when combined with exogenous copper ionophores. This elevation in copper concentrations induces mitochondrial proteotoxic stress, promoting cuproptosis and inhibiting tumor metastasis in gastric cancer. Our findings highlight PTBP3 as a crucial oncogenic splicing factor, and targeting the alternative splicing events regulated by PTBP3 could serve as an effective therapeutic strategy for peritoneal metastasis in gastric cancer.

## 2 Results

### 2.1 PTBP3 Overexpression in Cancer Cells of Gastric Cancer Peritoneal Metastases

**Figure 1:**
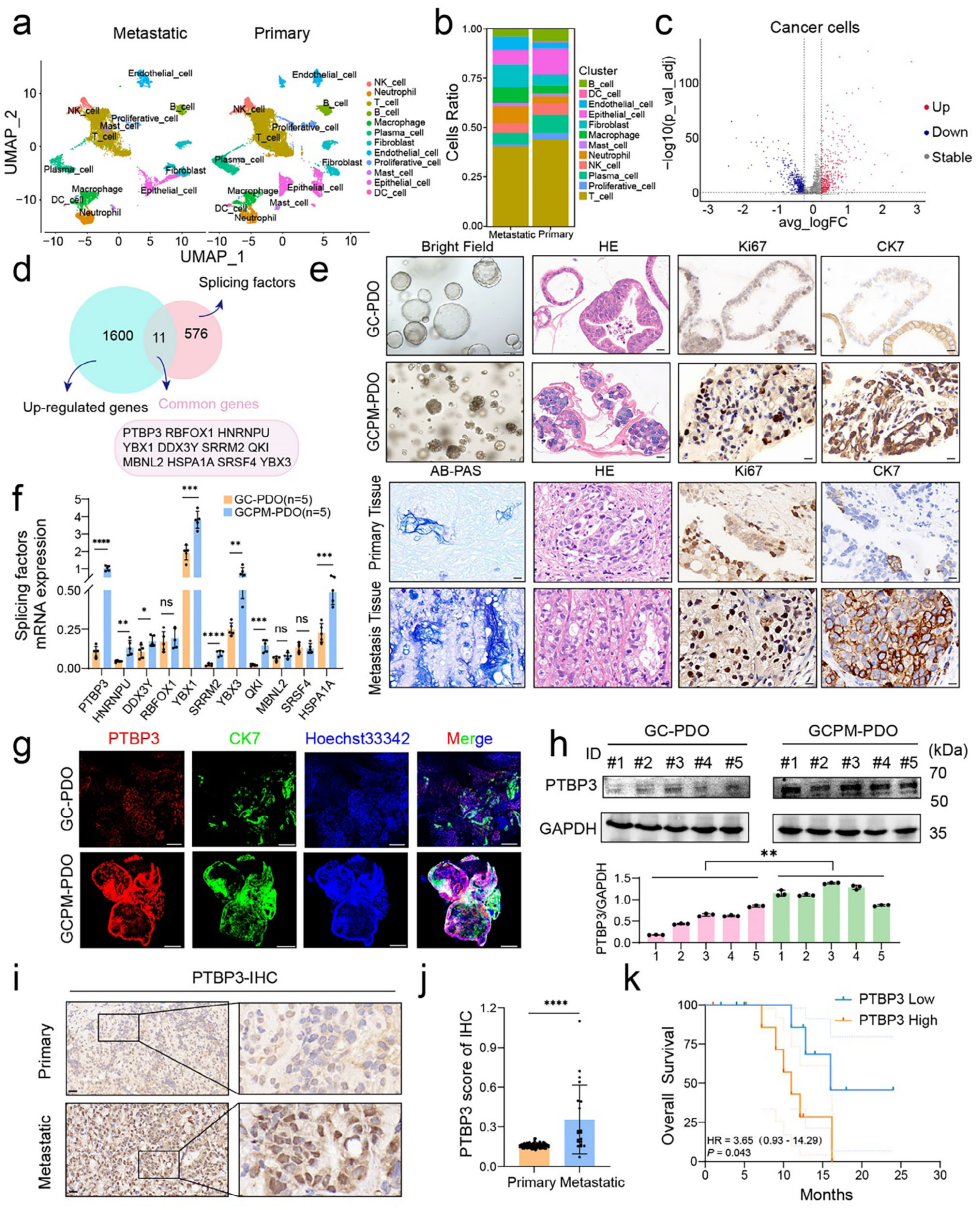
PTBP3 overexpression in cancer cells of gastric cancer peritoneal metastases. a) UMAP plots of three primary GC and three peritoneal metastasis GC samples from publicly available single‐cell datasets, grouped into 12 main cell types: NK cells, neutrophils, T cells, B cells, macrophages, plasma cells, fibroblasts, endothelial cells, proliferative cells, mast cells, epithelial cells, and DC cells. b) Proportion of each cell type in primary and metastatic samples. c) Volcano plot of gene expression in metastatic GC samples compared to primary GC samples (upregulated: *P* < 0.05, log(fold change) > 0.25; downregulated: *P* < 0.05, log(fold change) < 0.25). d) Venn diagram showing common splicing factors from the literature and genes with significant expression in the volcano plot. e) Bright‐field microscopy images of organoids derived from primary (top) and metastatic gastric cancer tissues (bottom), with H&E staining and immunohistochemical staining for Ki67 and CK7 (Scale bars, 100 µm for bright‐field, 20 µm for immunohistochemical staining). f) Histogram of mRNA expression levels of common genes in primary and metastatic organoids (*n* = 5). g) Representative fluorescence microscopy images of organoids showing double staining (red: PTBP3, green: CK7), with Hoechst33342‐stained nuclei (blue). Scale bars, 100 µm. h) Immunoblot analysis of PTBP3 in primary (*n* = 5) and metastatic organoids (*n* = 5), with GAPDH as loading control (top). Quantification of PTBP3 protein relative to GAPDH (bottom). i) Immunohistochemical staining of PTBP3 in primary (top) and metastatic (bottom) gastric cancer tissues. Scale bars, 100 µm. j) Quantification of PTBP3 immunohistochemistry scores in primary (*n* = 102) and metastatic (*n* = 20) tissues. k) Survival analysis based on PTBP3 expression levels in gastric cancer patients with peritoneal metastasis (*n* = 20), plotted using GraphPad. Data are expressed as mean ± s.d., *n* ≥ 3. Unpaired two‐tailed Student's *t*‐test (f, j). ns, no significant, *P < 0.05, **P < 0.01, ***P < 0.001, ****P < 0.0001.

To delineate the differentially expressed splicing factors between primary gastric tumors and peritoneal metastatic lesions, we analyzed publicly available single‐cell RNA sequencing datasets (GSE163558, GSE183904), selecting three peritoneal metastatic tissue samples and three primary tumor tissue samples for analysis. Using well‐established marker genes for cell subtype annotation, we performed Uniform Manifold Approximation and Projection (UMAP) dimensionality reduction to elucidate the cellular composition of both cohorts (**Figure** **1**a; Figures S1 and S2a, Supporting Information). We also quantitatively assessed the relative proportions of distinct cell populations (Figure 1b). InferCNV analysis was performed to identify malignant cells within the epithelial cell populations in both groups (Figure S2b,c, Supporting Information). Differentially expressed genes between the tumor cells of the two groups were visualized using a volcano plot (Figure 1c and Data S1, Supporting Information). We intersected this set of highly expressed genes with a common collection of splicing factors, resulting in the identification of 11 splicing factors (PTBP3, HNRNPU, DDX3Y, RBFOX1, YBX1, SRRM2, YBX3, QKI, MBNL2, SRSF4, HSPA1A) with elevated expression levels (Figure 1d). To investigate the cellular changes in gastric cancer during peritoneal metastasis, we successfully established organoid models derived from both primary gastric cancer tissues and peritoneal metastatic tissues. Notably, organoids derived from primary gastric cancer tissues exhibited a vacuolated morphology, whereas those derived from peritoneal metastatic tissues displayed tightly packed cellular arrangements, consistent with previously published studies.^[^ ^24^, ^25^ ^]^ Representative bright‐field images of the organoids were captured, and subsequent analyses included hematoxylin and eosin (H&E) staining as well as immunohistochemical analysis of key biomarkers, including Ki67 and CK7. In addition, AB‐PAS, H&E, Ki67, and CK7 immunohistochemical images of organoid‐derived tissues from both primary and metastatic tumor tissues were presented (Figure 1e). We provided expression indicators for gastric cancer primary tumor tissues and their corresponding organoids (Figure S2d, Supporting Information), as well as molecular characterization of an organoid derived from a metastatic lesion and its corresponding metastatic tissue (Figure S2e, Supporting Information). Ki67 and CK7 fluorescence double staining was performed on organoids derived from both primary and metastatic gastric tumors to better illustrate the morphology and expression profiles of organoids from different sources (Figure S2f, Supporting Information). RNA was extracted from the organoids, and the mRNA expression levels of splicing factors were quantitatively assessed across the two distinct organoid models. The expression levels of PTBP3, HNRNPU, DDX3Y, YBX1, SRRM2, YBX3, QKI, and HSPA1A were significantly higher in organoids derived from metastatic lesions compared to those derived from primary lesions (Figure 1f). In addition, the mRNA expression levels of these eleven splicing factors in metastatic and primary tumor tissues were also analyzed (Figure S2g, Supporting Information). The results indicated that, compared to primary tumor tissues, the expression levels of PTBP3, RBFOX1, SRRM2, YBX3, and QKI were significantly higher in metastatic tumor tissues. Among these splicing factors, the difference in PTBP3 expression was the most significant. Furthermore, by combining expression data from the Kaplan–Meier Plotter database with patient overall survival and progression‐free survival outcomes, we found that the expression levels of PTBP3, SRRM2, QKI, SRSF4, and YBX3 were statistically significant and consistent with patient overall survival and progression‐free survival. Higher expression levels were associated with poorer prognosis (Figures S3 and S4, Supporting Information). In conclusion, we believe that PTBP3 plays a crucial role in promoting peritoneal metastasis of gastric cancer, and therefore, we selected PTBP3 as the target molecule for further investigation. To determine the expression pattern of PTBP3 within the organoids, we performed immunofluorescence staining, enabling the precise localization of PTBP3 within the organoid structures (Figure 1g). Western blot analysis further confirmed that PTBP3 protein levels were significantly higher in organoids derived from metastatic tissues compared to those from primary tumors (Figure 1h and S5a, Supporting Information). To evaluate the clinical significance of PTBP3 in gastric cancer, we conducted immunohistochemical analysis using a tissue microarray comprising 102 pairs of matched gastric cancer normal tissues and primary tumors, alongside 20 cases of non‐paired gastric cancer peritoneal metastatic tissues (Figure 1i and Figure S5b, Supporting Information). Immunohistochemical staining revealed that PTBP3 was significantly upregulated in primary tumor tissues compared to adjacent normal tissues and exhibited significant overexpression in peritoneal metastatic lesions compared to primary tumors (Figure 1j). Survival analysis of PTBP3 expression in peritoneal metastases of gastric cancer revealed that high expression was associated with poorer prognosis (Figure 1k). Furthermore, clinical and pathological analysis of 102 primary gastric cancer cases demonstrated that high PTBP3 expression was positively correlated with both T stage and peritoneal metastasis, suggesting its potential role in disease progression (Figure S5c and Table S1, Supporting Information). Collectively, these findings demonstrate that the splicing factor PTBP3 is significantly upregulated in tumor cells of peritoneal metastatic lesions in gastric cancer. This overexpression is closely associated with adverse clinical outcomes, highlighting its prognostic value and potential as a therapeutic target.

### 2.2 PTBP3 Promotes the Invasion and Proliferation of Gastric Cancer Cells and Organoids

**Figure 2:**
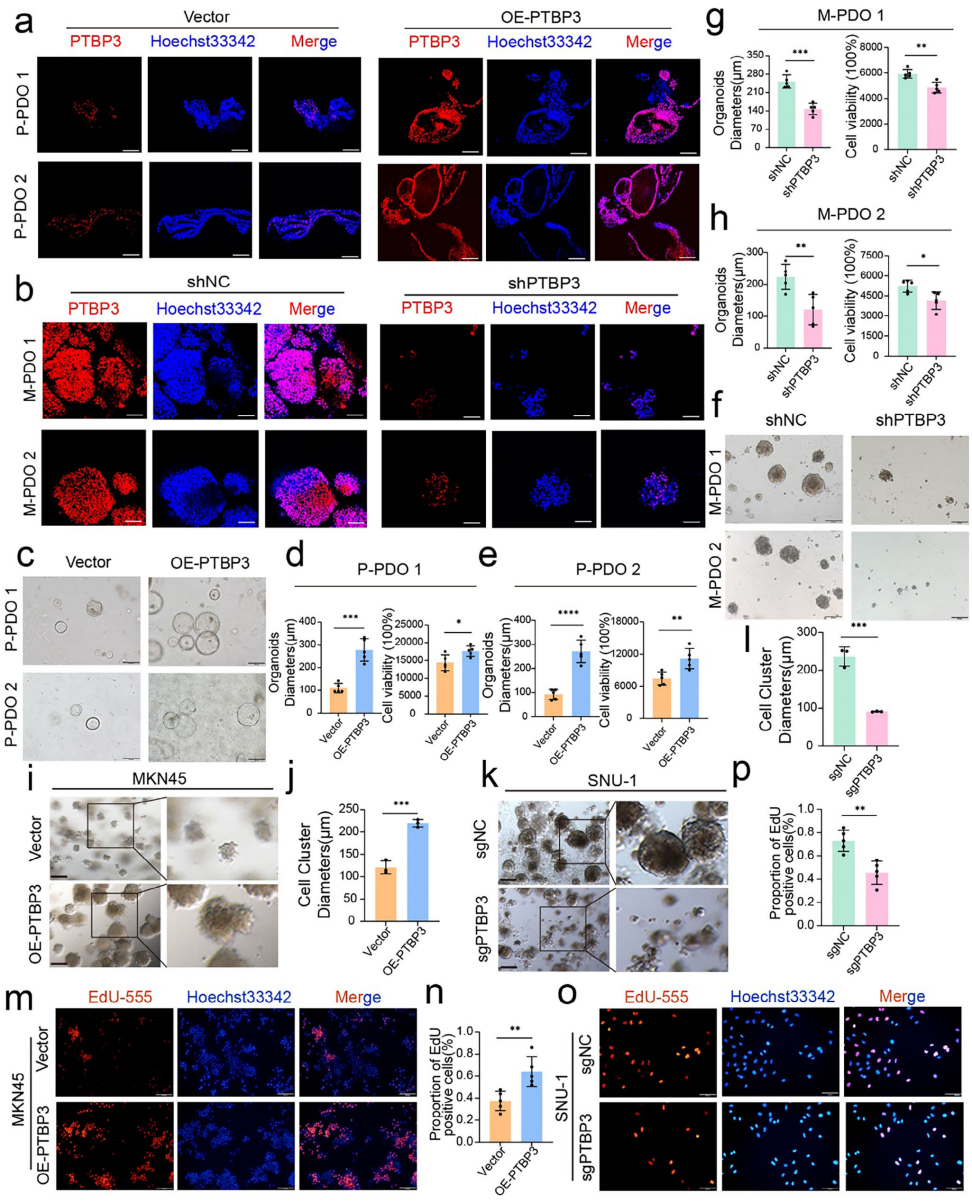
PTBP3 promotes the invasion and proliferation of gastric cancer cells and organoids. a) Representative immunofluorescence images of GC organoids with (right) or without (left) PTBP3 overexpression. Scale bars, 100 µm. b) Representative immunofluorescence images of GCPM organoids with (right) or without (left) PTBP3 knockdown. Scale bars, 100 µm. c–e) Representative bright‐field images, viability, and organoid diameters of GC organoids with (right) or without (left) PTBP3 overexpression (*n* = 5 for each group; data are mean ± s.d.). Scale bars, 200 µm. f–h) Representative bright‐field images, viability, and organoid diameters of GCPM organoids with (right) or without (left) PTBP3 knockdown (*n* = 5 for each group; data are mean ± s.d.). Scale bars, 200 µm. i) Bright‐field photograph of tumor cells growing as clusters in matrix glue. 3D matrigel outgrowth of PTBP3‐overexpressing MKN45 cells is enhanced. Left, normal field; right, magnified image. Scale bars, 200 µm. j) Histogram of spheroid diameters for two groups. The average diameter of spheres was calculated for each field (*n* = 3; data are mean ± s.d.). k) Bright‐field photograph of tumor cells growing as clusters in matrix glue. 3D matrigel outgrowth of PTBP3‐knockout SNU‐1 cells is impaired. Left, normal field; right, magnified image. Scale bars, 200 µm. l) Histogram of spheroid diameters for two groups. The average diameter of spheres was calculated for each field (*n* = 3; data are mean ± s.d.). m,n) EdU assay to assess the impact of PTBP3 overexpression on cell proliferation (*n* = 5 for each group; data are mean ± s.d.). Scale bars, 100 µm. o,p) EdU assay to assess the impact of PTBP3 knockout on cell proliferation (*n* = 5 for each group; data are mean ± s.d.). Scale bars, 100 µm. In all panels, data are expressed as mean ± s.d., *n* ≥ 3. Unpaired two‐tailed Student's *t*‐test (d, e, g, h, j, i, n, p). *P < 0.05, **P < 0.01, ***P < 0.001, ****P < 0.0001.

To elucidate the role of PTBP3 in gastric cancer tumor cells, we transduced organoids with lentiviral vectors to establish PTBP3‐overexpressing models in organoids derived from primary gastric cancer tissues (P‐PDO) and PTBP3‐knockdown models in organoids derived from peritoneal metastatic gastric cancer tissues (M‐PDO). PTBP3 expression levels in these organoids were validated through immunofluorescence assays and Western blot analysis (**Figure** **2**a,b and S6a, Supporting Information). PTBP3 mediated invasive growth and proliferation of organoids within the matrix gel, as shown by bright‐field images of organoids captured on day 7 post‐plating for both control and treatment groups. In organoids derived from primary tumors (Figure 2c), PTBP3 overexpression resulted in larger diameters and enhanced viability (Figure 2d,e). In contrast, in peritoneal metastatic gastric cancer organoids (Figure 2f), PTBP3 knockdown reduced organoid diameter and viability (Figure 2g,h). We also selected MKN45 cells for lentiviral infection to establish control and PTBP3‐overexpressing cell lines, and SNU‐1 cells for plasmid transfection to create control and PTBP3‐knockout cell lines, based on PTBP3 expression levels in commonly used gastric cancer cell lines (Figure S6b–d, Supporting Information). Western blot (WB) analysis and immunofluorescence assays were conducted to validate PTBP3 expression in each group, confirming successful overexpression and knockout (Figure S6e,f, Supporting Information). We performed a 3D matrix gel tumor spheroid assay using the established MKN45 (Figure 2i) and SNU‐1 (Figure 2k) cell lines to assess the impact of PTBP3 overexpression or knockout on spheroid invasive growth, quantifying spheroid diameters. Compared to the vector control group, PTBP3 overexpression significantly enhanced spheroid invasion potential (Figure 2j), while PTBP3 knockout inhibited tumor cell invasion, as indicated by a reduction in spheroid diameter (Figure 2l). We further evaluated the proliferative capacity of gastric cancer cells using the EdU assay. In MKN45 cells, overexpression of PTBP3 significantly increased the number of EdU‐positive cells (Figure 2m,n). Conversely, PTBP3 knockout in SNU‐1 cells reduced the number of EdU‐positive cells (Figure 2o,p). Transwell assays were also performed on MKN45 and SNU‐1 cell lines to investigate the effect of PTBP3 on cell migration and invasion (Figure S6g–j, Supporting Information). The results showed that PTBP3 overexpression in MKN45 cells enhanced migration and invasion, while PTBP3 knockdown in SNU‐1 cells inhibited these abilities. Taken together, these functional experiments confirm that PTBP3 mediates the invasive and proliferative activities of gastric cancer cells and organoids, thereby promoting gastric cancer metastasis.

### 2.3 PTBP3 Regulates Splicing Events during the Progression of Peritoneal Metastasis in Gastric Cancer

**Figure 3:**
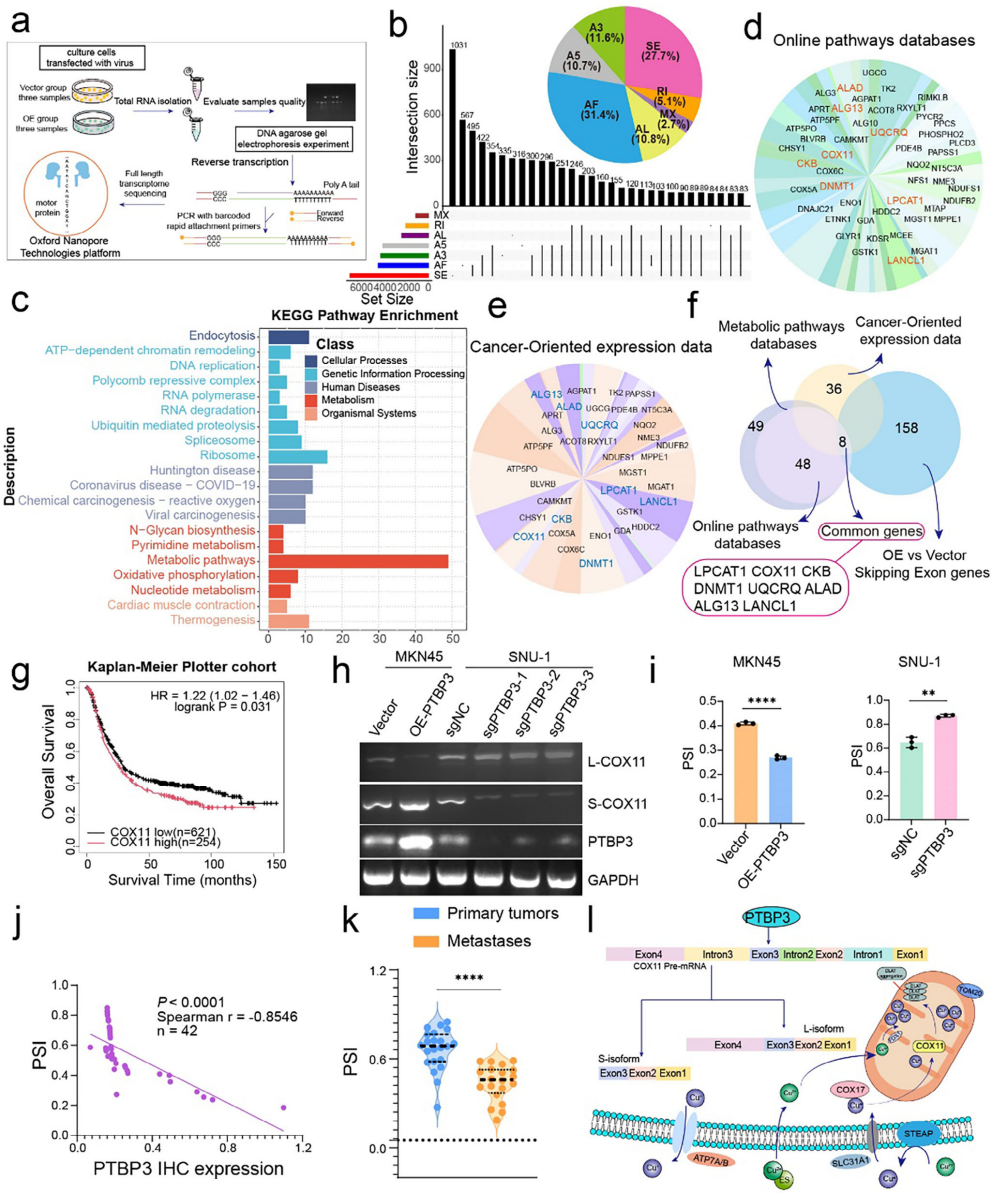
PTBP3 regulates splicing events during the progression of peritoneal metastasis in gastric cancer. a) Flowchart illustrating the preparation of samples and the process of full‐length transcriptome sequencing. b) Different types of AS events and their distribution based on the ONT long‐read transcriptome of our samples. c) KEGG pathway analysis of significant differentially regulated AS events between samples. d,e) Two additional public databases used to enrich important associated pathways, with major gene names labeled in pie charts. f) Venn diagram showing the overlap of genes from pathway analysis and those exhibiting exon skipping (*P* < 0.5, │∆PSI│ > 10%). g) Kaplan–Meier analysis of the correlation between COX11 expression and overall survival. h,i) PCR with reverse transcription (RT‐PCR) (left) and quantification of gray values for COX11 isoforms in SNU‐1 cells expressing CRISPR‐Cas9 control (sgNC), sgPTBP3‐1, sgPTBP3‐2, or sgPTBP3‐3, and MKN45 cells expressing vector or overexpressing PTBP3 (OE‐PTBP3) (right). j) Correlation analysis between PSI and PTBP3 IHC expression, showing a positive correlation as determined by Spearman analysis. k) Violin plot of COX11 PSI values in gastric cancer patient samples (*n* = 22 primary tumors and *n* = 20 metastatic tumors). l) Schematic diagram depicting PTBP3‐mediated conversion of long and short COX11 transcripts involved in copper ion homeostasis in gastric cancer. In all panels, data are expressed as mean ± s.d., *n* ≥ 3. Unpaired two‐tailed Student's *t*‐test (i, k). **P < 0.01, ****P < 0.0001.

To gain deeper insight into the molecular mechanisms by which PTBP3 orchestrates peritoneal metastasis in gastric cancer, we performed full‐length transcriptome sequencing on MKN45 cells with or without PTBP3 overexpression (**Figure** **3**a). This approach provided a comprehensive profile of splicing events regulated by PTBP3. The UpSet plot revealed that exon skipping and first exon skipping were the most prevalent types of alternative splicing (Figure 3b). These results suggest that PTBP3‐mediated alternative splicing regulation is highly diverse (Figure S7a, Supporting Information), providing a solid foundation for further investigation into its mechanisms in gastric cancer metastasis.

PTBP3 typically functions as a splicing repressor, influencing downstream gene alternative splicing, often leading to exon skipping. Consequently, we identified 158 genes exhibiting PTBP3‐dependent exon skipping events, selected based on P values less than 0.05 and |ΔPSI| greater than 10% (Data S2, Supporting Information). To elucidate the biological processes associated with the metastatic progression of gastric cancer cells mediated by PTBP3 splicing targets, we performed KEGG pathway enrichment analysis on gene sets exhibiting differential alternative splicing events. The results revealed that metabolic‐related signaling pathways represented a significant proportion of the enriched pathways (Figure 3c). In addition, we conducted GSEA enrichment analysis using gene sets from the MSigDB database, including C2 (curated gene sets from online pathway databases, publications in PubMed, and expert knowledge) and C4 (computational gene sets derived from large collections of cancer‐oriented expression data), on the differentially expressed genes from the two groups. We also performed KEGG pathway analysis on the differentially spliced genes to determine whether any tumor‐related pathways were enriched among the 49 metabolic‐related signaling pathways identified. The results of the GSEA enrichment analysis for C2 and C4 gene sets from MSigDB are presented as pie charts (Figure 3d,e), showing gene names and their proportions. Further, by intersecting the results of the above analyses, we identified the common genes (LPCA1, COX11, CKB, DNMT1, UQCRQ, ALAD, ALG13, LANCL1), which are the focus of our further investigation (Figure 3f).

Subsequently, GSEA was conducted on the signaling pathways associated with these genes and their alternative splicing events. We found that mitochondrial‐related signaling pathways (Figure S7b, Supporting Information) and tricarboxylic acid (TCA) cycle‐related pathways (Figure S7c, Supporting Information) played critical roles in this context. Moreover, GSEA analysis of differentially expressed genes between peritoneal metastatic lesions and primary tumors, based on initial single‐cell RNA sequencing data, revealed significant involvement of pathways related to copper ion transport and intracellular copper homeostasis (Figure S7d, Supporting Information).

Among the identified genes, COX11, localized in the mitochondrial inner membrane, plays a crucial role in regulating copper homeostasis. Recent studies have shown that excessive intracellular copper accumulation can lead to a specific form of cell death known as cuproptosis, which is increasingly recognized for its association with tumorigenesis and progression. Analysis using the Kaplan–Meier Plotter database indicated a negative correlation between high COX11 expression and overall survival in patients (Figure 3g), suggesting that COX11 could serve as a prognostic marker for poor clinical outcomes. Furthermore, PTBP3 is known to preferentially bind to pre‐mRNA regions enriched in cytosine (C) and uracil (U). To further investigate the potential regulatory role of PTBP3 in COX11 expression, we predicted its binding sites to assess the possibility of PTBP3‐mediated alternative splicing of COX11 (Figure S7e, Supporting Information). Therefore, we propose that COX11 holds significant research value in facilitating the malignant progression of gastric tumors and the peritoneal metastasis of gastric cancer.

To confirm whether COX11 undergoes exon skipping mediated by PTBP3, we first conducted agarose gel electrophoresis (AGE), which revealed two distinct transcripts—long and short forms—of the COX11 gene across various gastric cancer cell lines (Figure S8a, Supporting Information). Sanger sequencing further confirmed their sequence distribution (Figure S8b, Supporting Information). Next, we examined transcript levels of COX11 in MKN45 cells overexpressing PTBP3, observing a significant increase in the expression of the shorter transcript compared to the control group. In contrast, in SNU‐1 cells with PTBP3 knockout, the proportion of the shorter COX11 transcript decreased relative to the control group (Figure 3h,i). In addition, we assessed transcript changes of other relevant genes (LPCA1, CKB, DNMT1, UQCRQ, ALAD, ALG13, LANCL1). Based on annotations from the Ensembl database, only one protein is translated from the different transcripts of LPCAT1 and UQCRQ, so we did not investigate their transcript variations under PTBP3 regulation further (Figure S8c,d, Supporting Information).

We further examined the histological profiles of the long and short transcripts of COX11. A correlation analysis between the percent splicing index (PSI) values of COX11 and PTBP3 protein expression levels was conducted on 20 gastric cancer peritoneal metastatic samples and 22 primary gastric cancer samples, revealing a significant correlation between these factors (Figure 3j). Data from the Kaplan–Meier Plotter database also indicated a strong association between the overall expression levels of both genes in gastric cancer (Figure S9a, Supporting Information). We analyzed the PSI values of gastric cancer peritoneal metastatic samples compared to primary gastric cancer samples and found that the metastatic samples exhibited lower PSI values (Figure 3k and Figure S9b, Supporting Information). Moreover, increased expression levels of the shorter transcript correlated with poorer overall survival rates in patients (Figure S9c, Supporting Information). These findings suggest that COX11 exon skipping is more prevalent in gastric cancer peritoneal metastatic samples, leading to the generation of a higher abundance of shorter transcripts, which are associated with unfavorable patient prognosis.

Through our analyses, we determined that PTBP3 is a crucial splicing factor involved in the progression of gastric cancer peritoneal metastasis, participating in numerous alternative splicing events. It facilitates the alternative splicing of the downstream target gene COX11, a mitochondrial copper transport protein essential for maintaining intracellular copper homeostasis. Upon the introduction of exogenous copper ionophores, the concentration of copper ions within mitochondria increases. Under the influence of FDX1, divalent copper ions (Cu^2^⁺) are reduced to the more toxic monovalent copper (Cu⁺). COX11, through variations in its transcript lengths, regulates endogenous copper levels. When copper concentration within mitochondria increases, it triggers a proteotoxic stress response, thereby promoting cuproptosis. Thus, we propose that COX11 contributes to the induction of cuproptosis. We developed a schematic model to illustrate these relationships (Figure 3l) and further explored the connection between this process and gastric cancer peritoneal metastasis.

### 2.4 ASO Drugs Targeting S‐COX11 Attenuate the Proliferative and Invasive Capabilities of Gastric Cancer Cells and Organoids

ASOs are short DNA fragments, typically 10–20 nucleotides in length. Upon entering cells, they hybridize with target mRNA through complementary binding, which not only obstructs mRNA binding to ribosomes but also activates ribonuclease H (RNase H) to degrade the RNA–DNA hybrid duplex. This process effectively reduces target mRNA levels. Accordingly, we designed and synthesized 2′‐MOE‐modified ASOs based on the sequence of the short COX11 transcript (S‐COX11) (Figure S10a, Supporting Information) to investigate their impact by downregulating this transcript.

**Figure 4:**
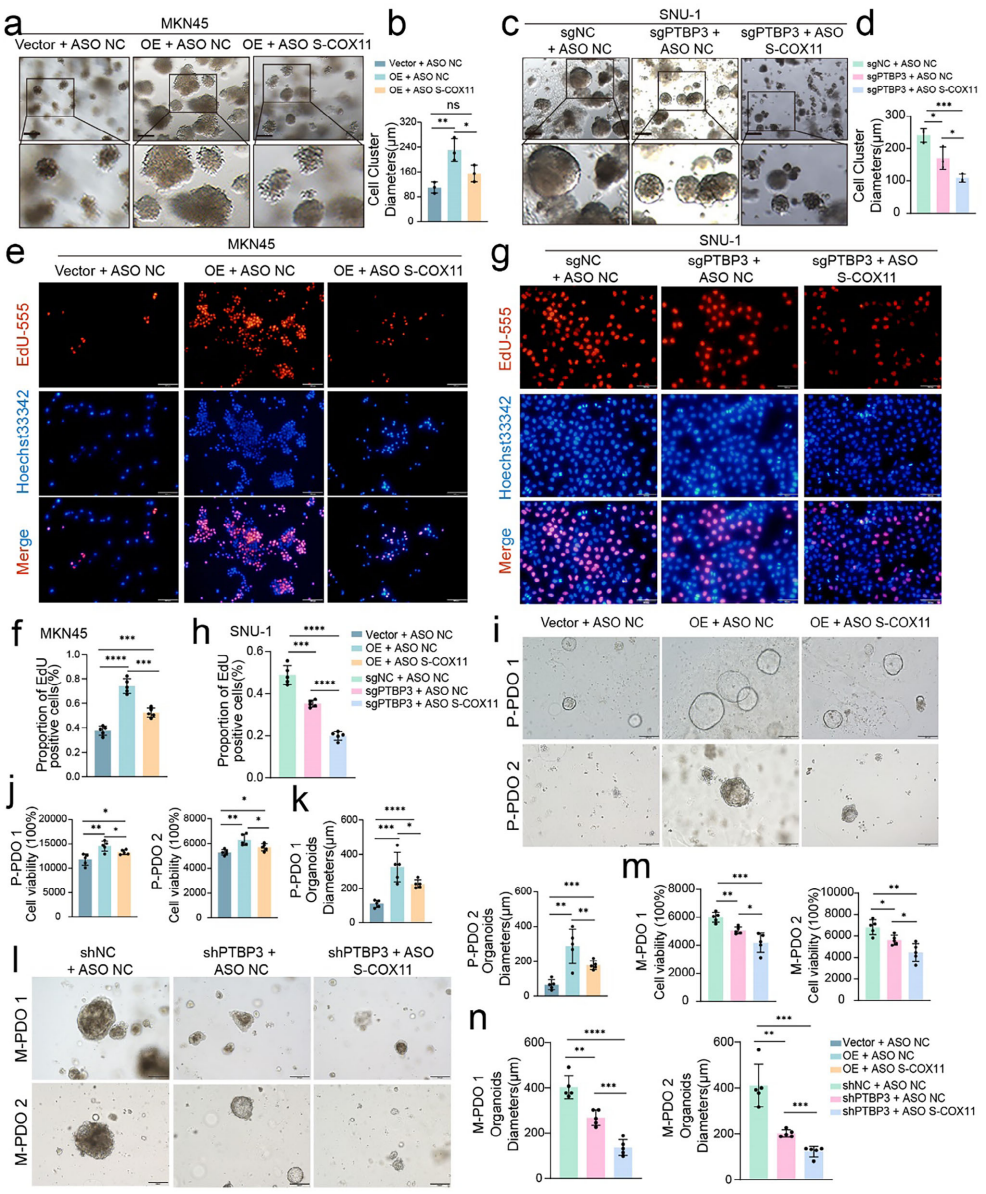
ASO drugs targeting S‐COX11 attenuate the proliferative and invasive capabilities of gastric cancer cells and organoids. a,b) Representative images (left) and quantitative comparison (right) of 3D matrigel spheroid formation in COX11 rescue assays in MKN45 cells. Top: normal field. Bottom: magnified image. Scale bars, 200 µm. c,d) Representative images (left) and quantitative comparison (right) of 3D matrigel spheroid formation in COX11 rescue assays in SNU‐1 cells. Top: normal field. Bottom: magnified image. Scale bars, 200 µm. e) Representative images of EdU assays in COX11 rescue assays in MKN45 cells. Scale bars, 100 µm. f) Quantitative comparison of EdU assays in COX11 rescue assays in MKN45 cells (*n* = 5 for each group; data are mean ± s.d.). g) Representative images of EdU assays in COX11 rescue assays in SNU‐1 cells. Scale bars, 100 µm. h) Quantitative comparison of EdU assays in COX11 rescue assays in SNU‐1 cells (*n* = 5 for each group; data are mean ± s.d.). i–k) Representative bright‐field images (left) and analysis of GC organoid viability and diameters (right) in COX11 rescue assays (*n* = 5 for each group; data are mean ± s.d.). Scale bars, 200 µm. l–n) Representative bright‐field images (left) and analysis of GCPM organoid viability and diameters (right) in COX11 rescue assays (*n* = 5 for each group; data are mean ± s.d.). Scale bars, 200 µm. In all panels, data are expressed as mean ± s.d., *n* ≥ 3. Unpaired two‐tailed Student's *t*‐test (b, d, f, h, j, k, m, n). ns, no significant, *P < 0.05, **P < 0.01, ***P < 0.001, ****P < 0.0001.

RT‐PCR experiments confirmed the inhibitory effect of the ASOs on COX11 transcript expression (Figure S10b, Supporting Information), with ASO 2 significantly reducing the production of the short COX11 transcript. Recovery experiments were performed by treating MKN45 cells overexpressing PTBP3 and SNU‐1 cells with PTBP3 knockout (Figure S10c, Supporting Information). The results indicated that downregulation of S‐COX11 expression in MKN45 cells significantly impaired their spheroid invasion capacity in Matrigel (**Figure** **4**a,b). Similarly, in SNU‐1 cells with PTBP3 knockout, silencing of S‐COX11 expression further inhibited tumor spheroid invasion (Figure 4c,d). In addition, EdU proliferation assays yielded consistent findings. In MKN45 cells, the addition of ASOs targeting S‐COX11 reduced the number of EdU‐positive cells compared to the control group with PTBP3 overexpression alone (Figure 4e,f). In SNU‐1 cells, treatment with ASOs targeting the short COX11 transcript further decreased the number of EdU‐positive cells relative to the PTBP3 knockout group (Figure 4g,h). In the Transwell experiment, we also observed that the addition of ASO S‐COX11 suppressed the migration and invasion abilities of gastric cancer cells (Figure S10e–h, Supporting Information). To further investigate whether the effects of PTBP3 on cell proliferation and migration depend on COX11, we inhibited S‐COX11 using siRNA in PTBP3‐overexpressing and knockdown cells. We repeated the 3D tumor cell Matrigel spheroid formation assay and tumor cell proliferation assays. The results demonstrated that overexpression of PTBP3 promoted tumor cell invasion (Figure S11a,b, Supporting Information) and proliferation (Figure S11e,f, Supporting Information), while knockdown of PTBP3 inhibited tumor cell invasion (Figure S11c,d, Supporting Information) and proliferation (Figure S11g,h, Supporting Information).

We also conducted recovery experiments on the organoids (Figure S10d, Supporting Information) to evaluate the effects of ASOs on the splicing regulation of S‐COX11 during organoid invasion and proliferation, by measuring organoid diameters and assessing viability changes. The results demonstrated that the addition of ASOs to organoids overexpressing PTBP3 significantly reduced organoid viability (Figure 4i) and compromised the invasive growth potential of organoids derived from primary gastric cancer tissues (Figure 4j,k). In organoids derived from metastatic lesions, ASOs targeting S‐COX11 further inhibited invasive growth capacity and diminished organoid viability compared to PTBP3 knockdown alone (Figure 4l–n). These recovery experiments collectively demonstrated that alterations in the long and short COX11 transcripts, mediated by PTBP3, significantly influence the invasion and proliferation of tumor cells and organoids. Targeting S‐COX11 with ASOs effectively suppresses tumor invasion and proliferation, thereby reducing gastric cancer peritoneal metastasis.

### 2.5 PTBP3‐Regulated Increase in S‐COX11 Enhances Gastric Cancer Peritoneal Metastasis in Mice

To explore the involvement of PTBP3 in modulating the alternative splicing of COX11 in vivo, we constructed MKN45 cells expressing luciferase, enabling the monitoring of changes in tumor behavior. Through lentiviral transfection, we achieved PTBP3 overexpression in these modified cells, allowing us to assess the impact of PTBP3 on COX11 splicing events and their implications for tumor biology.

**Figure 5:**
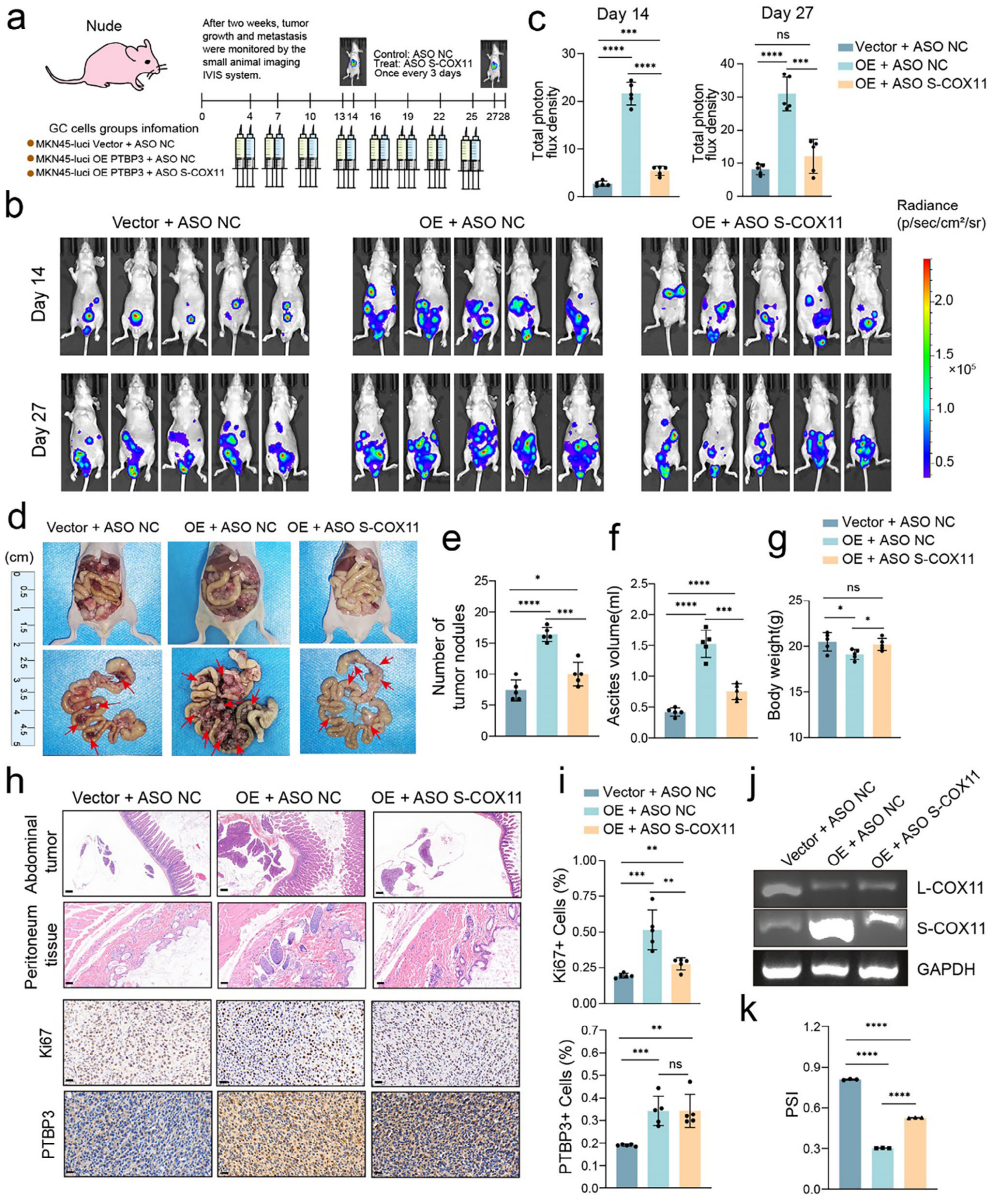
PTBP3‐regulated increase in S‐COX11 enhances gastric cancer peritoneal metastasis in mice. a) Schematic diagram showing the injection of MKN45 cells transfected with luciferase virus in combination with ASO drugs targeting the short isoform of COX11 in GC intraperitoneal injection models. b,c) MKN45 luciferase‐expressing cells transfected with PTBP3 overexpression lentivirus or vector lentivirus were intravenously injected into nude mice (*n* = 5 for each group). After one week, ASO drugs were administered for treatment. Bioluminescence intensity was monitored and total photon flux quantified at 14 and 27 d post‐injection. d) Representative images of GC tumors in the peritoneal implantation model following ASO drug treatment in each group. e) Quantification of the number of peritoneal tumor nodules at the end of the experiment (*n* = 5; data are mean ± s.d.). f) Quantification of the volume of bloody ascites at the end of the experiment (*n* = 5; data are mean ± s.d.). g) Quantification of the weight of mice at the end of the experiment (*n* = 5; data are mean ± s.d.). h,i) Representative H&E images of metastatic tumors implanted in the peritoneal cavity (upper) and paired peritoneal tissues (lower). Scale bars, 20 µm. Representative images (left) and quantification analysis (right) of Ki‐67 (upper) and PTBP3 (lower) IHC staining (*n* = 5; data are mean ± s.d.). Scale bars, 20 µm. j,k) RT‐PCR analysis of the two isoforms of PTBP3 in different groups (upper), with semi‐quantitative analysis of the strip gray values (lower). PSI = splice_in gray value / (splice_in gray value + splice_out gray value). In all panels, data are expressed as mean ± s.d., *n* ≥ 3. Unpaired two‐tailed Student's *t*‐test (c, e–g, i, k). ns, no significant, *P < 0.05, **P < 0.01, ***P < 0.001, ****P < 0.0001.

We injected the established MKN45 cells into the peritoneal cavity of nude mice and initiated ASO drug treatment on day 4 post‐injection (**Figure** **5**a). Subsequent drug interventions were administered every three days, and the luminescence intensity of the luciferase signal from the peritoneal tumors was monitored on days 14 and 27 to evaluate tumor development and treatment efficacy. The results demonstrated that MKN45 cells overexpressing PTBP3 exhibited increased tumorigenicity in the peritoneal cavity compared to the control group. However, ASO treatment significantly mitigated the tumorigenic potential of these cells. Notably, fluorescence intensity on days 14 and 27 in the ASO treatment group was markedly lower compared to the overexpression group (Figure 5b,c). Furthermore, we assessed tumorigenesis in the peritoneal cavity of the mice and performed pathological evaluations. Tumor cells were detected in both the peritoneum and abdominal organs, with the PTBP3 overexpression group exhibiting the highest number of tumor nodules. Importantly, ASO treatment led to a significant reduction in the number of tumor nodules (Figure 5d,e). In addition, the PTBP3 overexpression group experienced increased ascites, which was alleviated following ASO administration (Figure 5f).

Mice overexpressing PTBP3 exhibited weight loss due to aggressive tumor progression; however, this weight loss was alleviated after ASO treatment (Figure 5g). Immunohistochemical staining of animal tissues revealed a significant reduction in tumor cell proliferation, as indicated by Ki67 staining, following drug treatment compared to the overexpression group. PTBP3 expression was highest in the overexpression group, and ASO treatment did not significantly affect its expression (Figure 5h,i). We also analyzed the levels of long and short COX11 transcripts in animal tissues. The expression of the short COX11 transcript was significantly increased in the PTBP3 overexpression group, while ASO treatment effectively reduced S‐COX11 levels, resulting in notable changes in the PSI between the groups (Figure 5j,k).

These experimental results confirm that in the established animal model of peritoneal metastasis from gastric cancer cells, PTBP3 regulates COX11 alternative splicing, significantly impacting tumor metastatic progression. Furthermore, the ASO designed to target S‐COX11 demonstrated effective therapeutic potential in vivo.

### 2.6 ASO Drugs Targeting S‐COX11 Increased Endogenous Copper Concentrations

Elesclomol facilitates the transport of exogenous copper ions into cells, thereby increasing intracellular copper concentrations. It is often used in combination with copper chloride (CuCl_2_) to further elevate intracellular copper levels. Research indicates that excessive intracellular copper ions can induce a unique form of cell death known as cuproptosis, which diverges from traditional mechanisms of cell death and holds therapeutic potential for tumors. COX11, a copper transport protein located in the mitochondrial inner membrane, exhibits transcript changes regulated by the upstream gene PTBP3 in the context of peritoneal metastasis. Our goal is to determine whether ASO targeting the shorter isoform of the COX11 transcript can influence copper concentrations in mitochondria.

**Figure 6:**
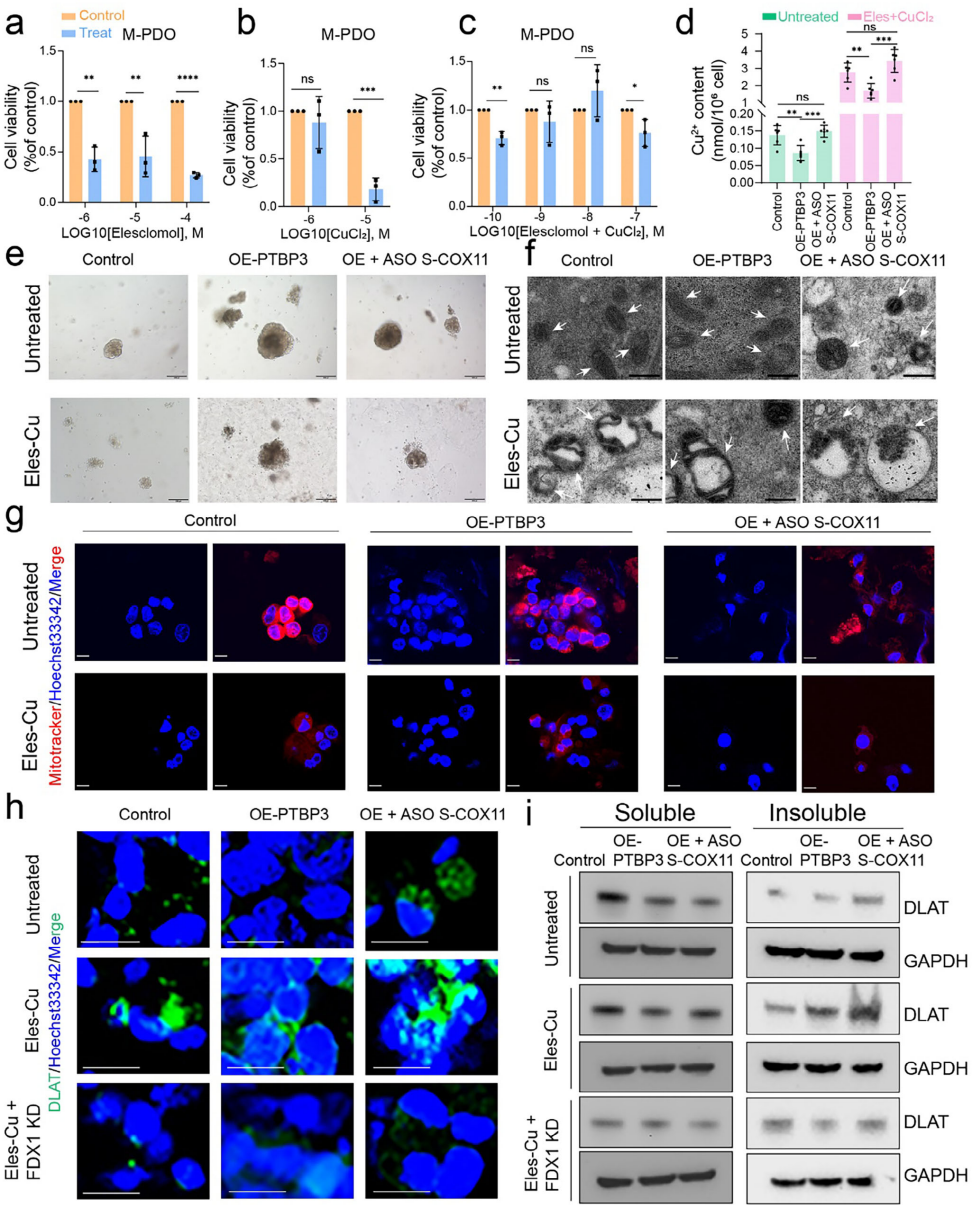
ASO drugs targeting S‐COX11 increased endogenous copper concentrations. a) Viability of organoids was assessed by adding different concentrations of Eles (elesclomol) for 24 h. b) Viability of organoids was assessed by adding different concentrations of CuCl_2_ for 24 h. c) Viability of organoids was assessed after treatment with Eles‐Cu (1:1 ratio) combination for 24 h. d) Bar graph showing the measured concentration of copper ions (Cu^2^⁺) in mitochondria. Left, untreated; right, treated with Eles‐Cu combination at a drug concentration of 10^−^⁸ m. e) Representative bright‐field images of organoids. Upper, untreated; lower, treated with Eles‐Cu combination at a drug concentration of 10^−^⁸ m after 3 d. Scale bars, 200 µm. f) Representative images of mitochondrial electron microscopy. Upper, untreated; lower, treated with Eles‐Cu combination at a drug concentration of 10^−^⁸ m after 3 d. Scale bars, 0.5 µm. g) Mitochondria were labeled with a mitochondrial probe and observed under a confocal microscope (red, Mitotracker; blue, Hoechst33342). Upper, untreated; lower, treated with Eles‐Cu combination at a drug concentration of 10^−^⁸ m after 3 d. Scale bars, 10 µm. h. DLAT protein oligomerization was analyzed by immunofluorescence (IF) (green, DLAT; blue, Hoechst33342). Upper, untreated; lower, treated with Eles‐Cu combination at a drug concentration of 10^−^⁸ m after 3 d. Scale bars, 30 µm. i) Western blot analysis was used to validate the expression of DLAT protein oligomerization in different samples after 24 h. In all panels, data are expressed as mean ± s.d., *n* ≥ 3. Unpaired two‐tailed Student's *t*‐test (a–d). ns, no significant, *P < 0.05, **P < 0.01, ***P < 0.001, ****P < 0.0001.

We utilized organoids derived from gastric cancer peritoneal metastases to assess the effects of varying concentrations of Elesclomol and CuCl₂ on organoid viability. After 24 h of treatment, we measured changes in organoid viability (**Figure** **6**a,b). Notably, even at the minimal concentration of Elesclomol (10^−6^ m), there was a substantial inhibition of organoid growth, whereas CuCl_2_ exhibited inhibitory effects at a higher concentration of 10^−5^ m. In subsequent experiments involving combination treatment, it was observed that at lower concentrations (10^−10^ m), the combination therapy effectively suppressed growth, while no significant effects were noted at concentrations of 10^−9^ and 10^−8^ m. Upon increasing the concentration of the combination treatment to 10^−7^ M, a marked inhibitory effect was again observed (Figure 6c). To mitigate any potential toxicity from the drugs affecting our results, we selected a combined treatment concentration of 10^−8^ m (10 nmol L^−1^) for further investigation into the changes associated with fluctuations in copper ion concentrations. The organoids were classified into three groups: a control group, a PTBP3‐overexpressing group, and a group overexpressing PTBP3 while receiving ASO targeting S‐COX11 treatment. We concurrently evaluated the impact of combined therapy with Elesclomol and CuCl_2_ on each group.

We initially assessed the concentrations of mitochondrial copper ions in each organoid group using a copper ion detection assay. The results revealed that, in the absence of Elesclomol and copper chloride (CuCl_2_), the group overexpressing PTBP3 exhibited marginally lower copper concentrations compared to the control group. In contrast, the group treated with ASO targeting S‐COX11 showed a noticeable increase in copper concentrations relative to the overexpression group. Importantly, following supplementation with Elesclomol and CuCl_2_, the changes in copper concentrations in the ASO‐treated group became significantly more pronounced (Figure 6d). Based on these findings, we hypothesize that the elevation of copper concentrations within mitochondria may be linked to cuproptosis.

To explore the potential effects of these changes, we extended the drug treatment duration to 72 h and performed bright‐field imaging and electron microscopy on the organoids to assess any alterations in external morphology and internal microstructure. Mitochondrial probes and immunofluorescence techniques were used to label mitochondria and related markers, enabling us to further investigate the relationship between ASO treatment‐mediated changes in S‐COX11 transcripts and variations in mitochondrial copper ion concentrations. Bright‐field images of the organoids showed that in those derived from metastatic lesions, overexpression of PTBP3 significantly enhanced organoid growth, while the introduction of ASO targeting S‐COX11 resulted in a marked suppression of growth. The combination treatment with Elesclomol and CuCl_2_ exerted a broad inhibitory effect on organoid development, with the most pronounced suppression observed in the ASO treatment group (Figure 6e).

Mitochondrial electron microscopy revealed no significant abnormalities in the PTBP3‐overexpressing group compared to the control group in the absence of Elesclomol and CuCl_2_, with intact mitochondrial structures observed. In contrast, the ASO drug treatment group exhibited alterations in mitochondrial morphology, including reduced mitochondrial lengths and occasional vacuolation. After treatment with Elesclomol and CuCl_2_, more pronounced vacuolation in mitochondria was observed in both the control and overexpression groups. However, in the ASO treatment group, the dual stimulation by ASO and copper ionophores was associated with evident signs of mitochondrial autophagy, suggesting that the cells may have experienced toxicity or damage (Figure 6f).

Fluorescence imaging using a mitochondrial‐specific probe demonstrated that, in the absence of Elesclomol and CuCl_2_, both the overexpression and control groups exhibited intense red fluorescence, indicative of structurally intact mitochondria. In the ASO‐targeting S‐COX11 treatment group, fluorescence intensity was slightly diminished, and mitochondrial arrangement appeared disordered. Following treatment with Elesclomol and CuCl_2_, overall fluorescence intensity decreased, indicating mitochondrial damage. The overexpression group still displayed mitochondria arranged in an orderly manner, whereas the ASO treatment group exhibited the weakest fluorescence intensity, with significant depletion of fluorescence, suggesting diminished mitochondrial activity and potential mitochondrial damage (Figure 6g). Immunofluorescence analysis of the mitochondrial membrane protein TOM20 also indirectly reflects the mitochondrial state in response to the treatments (Figure S12a, Supporting Information).

To further investigate whether copper toxicity induces mitochondrial cuproptosis, we utilized immunofluorescence techniques to assess the expression levels of oligomerization of dihydrolipoamide S‐acetyltransferase (DLAT), a key intermediate in cuproptosis (Figure 6h). In the untreated control group, DLAT expression was weak, and overexpression of PTBP3 did not result in DLAT oligomerization. However, following ASO targeting S‐COX11 treatment, there was a notable increase in oligomerized DLAT protein levels. Upon stimulation with Elesclomol and CuCl₂, both the overexpression and control groups exhibited elevated DLAT oligomerization, with the most pronounced increase occurring in the group receiving ASO drugs combined with the copper ionophore, as indicated by intense green fluorescence. Further validation of DLAT protein oligomerization in each group was performed through Western blot (WB) analysis. The addition of ASO treatment led to an increase in insoluble DLAT protein, indicating oligomerization. When co‐medication was applied, the insoluble DLAT protein content increased even further. However, when FDX1 was knocked down in organoids prior to drug treatment, reduced levels of DLAT protein were detected in both groups. These findings suggest that ASO treatment can elevate copper ion levels in mitochondria, and that the resulting protein oligomerization can be inhibited by FDX1 (Figure 6i). In addition, we examined the expression levels of lipoylated DLAT (Figure S12b, Supporting Information) and found that after treatment with ASO S‐COX11 or Elesclomol and CuCl_2_, lipoylated DLAT expression was reduced, with the lowest expression observed when the drugs were used in combination. Upon knockdown of FDX1, the reduction in lipoylated DLAT expression was even more pronounced. These findings indicate that alterations in the COX11 transcript play a pivotal role in regulating intracellular copper levels. Furthermore, the addition of exogenous copper ionophores amplifies this effect, promoting cuproptosis. Given this, S‐COX11 emerges as a potential therapeutic target for the modulation of tumor metastasis.

### 2.7 ASO Drugs Targeting S‐COX11 Combined with Elesclomol Induces Cuproptosis in the PDOX Model

To further validate the effects of ASO drugs and copper ionophores in vivo, we established a patient‐derived organoid‐based xenograft (PDOX) model. In this model, tumors derived from established organoids were implanted into immunodeficient mice, serving as a robust platform for the in vivo validation of the organoid system. Tumor tissues obtained post‐tumorigenesis exhibited a high degree of similarity in molecular markers and functional characteristics to those of the original tumor, making it an excellent model for evaluating the efficacy of personalized therapies.^[^ ^26^ ^]^

**Figure 7:**
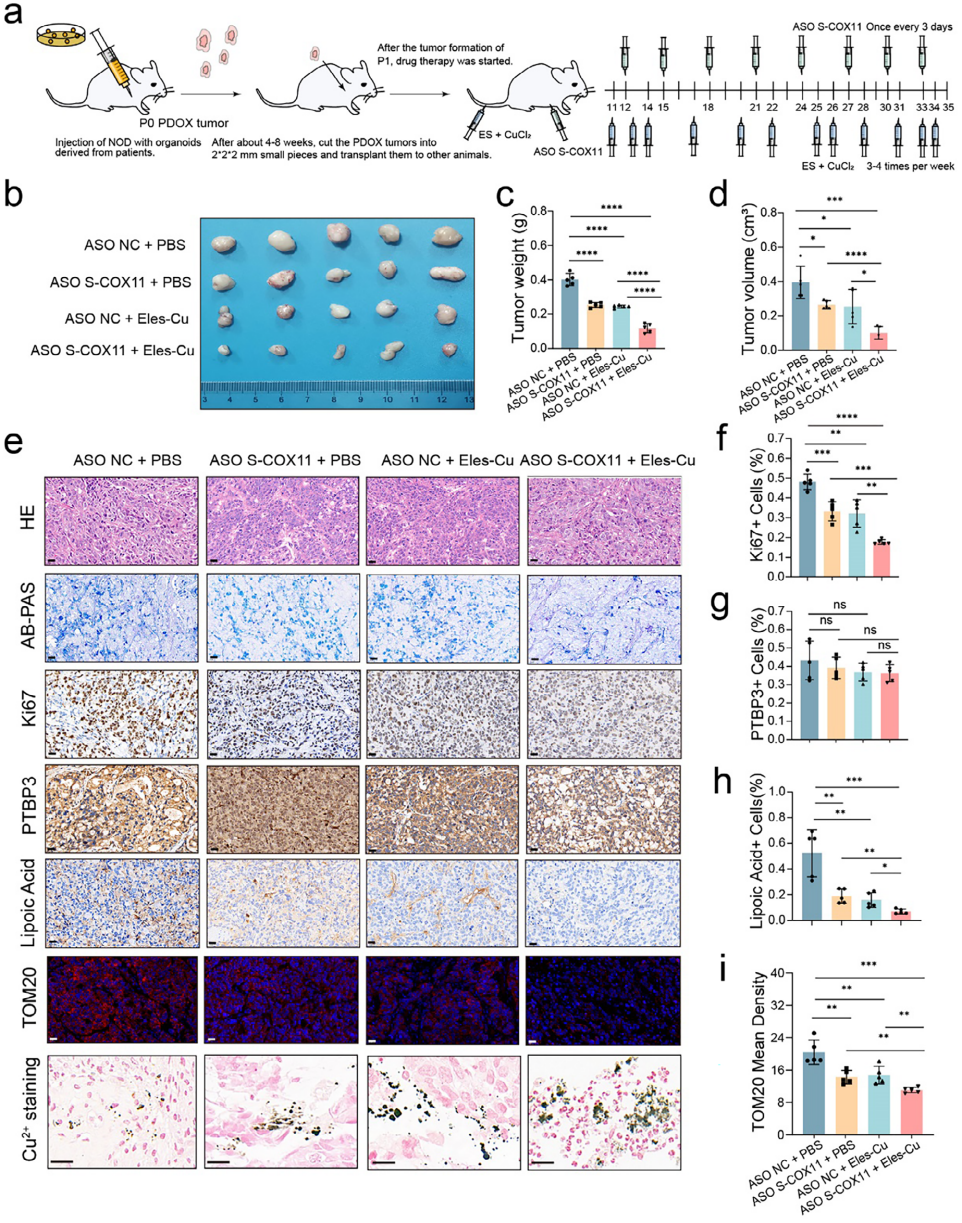
ASO drugs targeting S‐COX11 combined with Elesclomol induces cuproptosis in the PDOX model. a) Schematic diagram illustrating the treatment of GCPM PDOX mouse models with ASO drugs in combination with Eles (20 mg kg^−1^) and CuCl_2_ (0.05 mg kg^−1^). b) Representative images of PDOX tumors following treatment with either ASO drugs, Eles‐CuCl_2_, or their combination (*n* = 5 mice per group). c,d) Quantification of tumor weight (left) and tumor volume (right) in different groups at the end of mono‐ or combinational treatment. e) Representative images of H&E, AB‐PAS, Ki‐67 IHC staining, PTBP3 IHC staining, lipoic acid IHC staining, TOM20 immunofluorescence staining, and Cu^2^⁺ staining in GCPM PDOX tumors following mono‐ or combinational treatment. Scale bars, 20 µm. f–i) Quantification of Ki‐67 IHC staining, PTBP3 IHC staining, lipoylated protein IHC staining, and TOM20 immunofluorescence staining in GCPM PDOX tumors following mono‐ or combinational treatment (*n* = 5 per group). In all panels, data are expressed as mean ± s.d., *n* ≥ 3. Unpaired two‐tailed Student's *t*‐test (c, d, f–i). ns, no significant, *P < 0.05, **P < 0.01, ***P < 0.001, ****P < 0.0001.

Initially, we successfully established organoids in immunodeficient mice and harvested P0 generation tumor tissue for analysis of relevant indicators (Figure S13a, Supporting Information). Subsequently, P1 generation tumor transplantation experiments were conducted in similar immunodeficient mice. Tumor establishment was followed by drug treatment initiation approximately two weeks later to assess its impact on tumorigenesis and overall animal health, with the study concluding at the fifth week (**Figure** **7**a). In this study, ASO drugs were administered via intratumoral injection at a dose of 5 nmol every three days. Elesclomol (20 mg kg^−1^) was combined with copper chloride (0.05 mg kg^−1^) and delivered via tail vein injections, administered three to four times per week. The experimental design included four distinct groups: a control group, a group treated with ASO drugs alone, a group treated with Elesclomol and CuCl_2_, and a group treated with a combination of ASO drugs, Elesclomol, and copper chloride.

After the experiment, tumors were harvested from each group. Gross analysis revealed that treatment with ASO drugs alone or in combination with Elesclomol and CuCl_2_ effectively suppressed tumor growth. Compared to the untreated group, both tumor weight and volume were significantly reduced, with more pronounced effects observed in the combination treatment group (Figure 7b–d). Histological analysis of organoid tumorigenesis, performed using H&E and alcian blue‐periodic acid‐Schiff (AB‐PAS) staining, revealed key histopathological features (Figure 7e). Further analysis using Ki67 immunohistochemistry (IHC) indicated that both ASO drugs alone and the combination of Elesclomol with CuCl_2_ effectively inhibited tumor growth, with the most substantial suppressive effect observed in the group treated with ASO drugs combined with copper ionophores (Figure 7e,f). No significant differences in PTBP3 expression were observed among the four groups, given that the organoids originated from gastric cancer peritoneal metastatic lesions (Figure 7e,g).

Immunohistochemical and fluorescence analyses were performed to assess mitochondrial protein markers. Results revealed that lipoylated proteins were most abundant in untreated tumor tissues. In contrast, the group treated with a combination of ASO drugs, Elesclomol, and CuCl_2_ exhibited a significant reduction in lipoylated protein levels, as evidenced by diminished immunohistochemical staining compared to the monotherapy groups. This suggests that elevated copper levels in the tissue induced the formation of lipoylated protein oligomers, impairing normal physiological functions by reducing proteins entering the TCA cycle. Western blot analysis of lipoylated DLAT protein expression levels further validated this finding, showing a significant decrease in protein content in the combination therapy group, indicating the formation of lipoylated DLAT oligomers and the induction of mitochondrial cuproptosis (Figure 7e,h and Figure S13b, Supporting Information). Fluorescence staining for the mitochondrial protein TOM20 indicated that it was normally abundant on the outer mitochondrial membrane. However, in groups treated with ASO drugs alone or in combination with Elesclomol and CuCl_2_, elevated copper ion concentrations caused mitochondrial damage, leading to a notable decrease in TOM20 fluorescence. This effect was most significant in the combined treatment group, suggesting exacerbated mitochondrial injury and confirming that the combined therapy induced cellular cuproptosis (Figure 7e,i).

In addition, copper levels in the tissues were assessed using the rhodamine copper staining method. Compared to the untreated group, samples treated with ASO drugs alone or in combination with Elesclomol and copper chloride exhibited a significant increase in staining intensity, with the combined treatment group showing the most pronounced dark green coloration. This suggests a substantial accumulation of copper ions in the tissues of these animals (Figure 7e). Furthermore, analysis of COX11 transcript variants revealed that the expression of the long transcript (L‐COX11) was lower than that of the short transcript (S‐COX11), indicating a higher expression of S‐COX11 in the metastatic samples. After treatment with ASO targeting S‐COX11, the expression of S‐COX11 decreased significantly, while no substantial changes were observed in L‐COX11 levels (Figure S13c, Supporting Information).

These results demonstrate that in the PDOX model, the ASO drug targeting S‐COX11, in combination with Elesclomol and CuCl_2_, significantly increases intracellular copper ion concentrations. This, in turn, enhances the expression of lipoylated DLAT oligomers, thereby triggering cuproptosis and suppressing the progression of metastatic tumors.

## 3 Discussion

**Figure 8:**
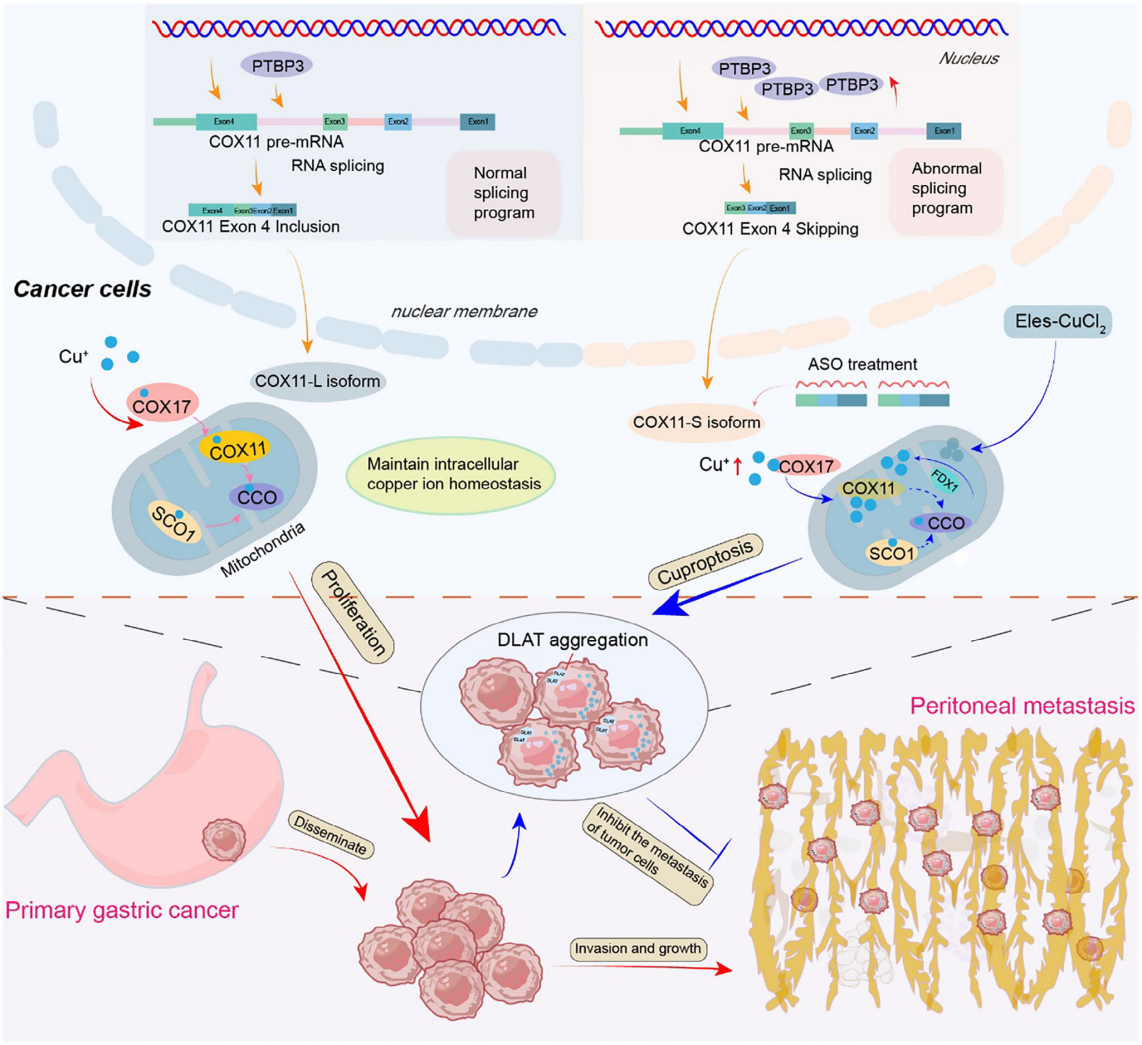
A schematic diagram illustrating the role of PTBP3 in gastric cancer peritoneal metastatic tissue. The expression level of PTBP3 is significantly elevated, which mediates the alternative splicing of the downstream gene COX11. This leads to aberrant splicing and the generation of shorter COX11 transcripts that are involved in regulating copper homeostasis within tumor cells. PTBP3 promotes tumor invasion and proliferation, facilitating the progression of gastric cancer from the primary site to metastatic seeding. The combined effects of the exogenous copper ionophore Elesclomol and copper chloride, in conjunction with ASO drugs targeting the shorter COX11 transcripts, further increase intracellular copper ion levels. This, in turn, activates cellular cuproptosis, contributing to the therapeutic efficacy against gastric cancer peritoneal metastasis.

Dysregulated expression of splicing factors and aberrant splicing processes have been implicated in tumorigenesis. Utilizing published single‐cell RNA sequencing data, we identified survival‐related splicing factors in peritoneal metastases of gastric cancer. Our findings show that PTBP3 is frequently upregulated in metastatic lesions, with elevated levels correlating with poor clinical outcomes. Further investigations revealed that PTBP3 promotes invasive growth in gastric cancer cells and organoids, driving tumor progression in a peritoneal injection model of gastric cancer cells. Notably, PTBP3 regulates COX11 alternative splicing, facilitating cuproptosis when combined with exogenous copper ionophores and ASO drugs targeting S‐COX11. These results underscore the oncogenic role of PTBP3 and suggest that targeting PTBP3‐mediated COX11 splicing regulation may provide a promising therapeutic approach to inhibit peritoneal metastasis in gastric cancer (**Figure** **8**).

Recent advancements in single‐cell sequencing technology have significantly enhanced our understanding of gene expression at the cellular level within tumor tissues. These innovations have provided detailed insights into cellular composition, the relative proportions of different cell types, intergroup differences, and the functional roles of tumor cells, as well as enabling molecular tumor subtyping. The spliceosome, the machinery responsible for alternative splicing in humans, is composed of essential spliceosome‐associated protein complexes and RNA. Cryo‐electron microscopy advancements have greatly improved our ability to predict and decipher the structural configuration of the spliceosome, offering profound insights into its architecture and function. Our research has shown that the splicing factor PTBP3 is overexpressed in gastric cancer peritoneal metastasis tissues. Bioinformatics analyses, supported by immunohistochemical validation of clinical specimens, confirmed this finding and established a correlation between high PTBP3 expression and poorer prognosis in patients. Data from public genomic databases further indicate that elevated PTBP3 expression is associated with unfavorable clinical outcomes. Functional analyses revealed that PTBP3 enhances invasion and proliferation in cellular and organoid models. In vivo experiments further demonstrated that PTBP3 promotes tumor growth and metastasis in the peritoneal cavity. PTBP3 is highly expressed in many cancers and is often associated with poorer survival outcomes. In gallbladder cancer, PTBP3 facilitates exon skipping in the IL‐18 gene, leading to the production of a variant isoform, ΔIL‐18, which is implicated in immune evasion mechanisms.^[^ ^27^ ^]^ In colorectal cancer, ovarian cancer, and endometrial cancer, PTBP3 phosphorylation is increased and correlates with cancer‐associated fibroblasts.^[^ ^28^ ^]^ In glioblastoma, PTBP3 is regulated by miR‐210, which controls glioblastoma cell proliferation and apoptosis,^[^ ^29^ ^]^ and PTBP3 also stabilizes Twist1, promoting tumorigenesis.^[^ ^30^ ^]^ In lung squamous cell carcinoma, PTBP3 regulates the cell cycle to promote cell proliferation^[^ ^31^ ^]^ and mediates TGF‐β‐induced epithelial–mesenchymal transition (EMT) and metastasis in lung adenocarcinoma cells.^[^ ^32^ ^]^ In gastric cancer, PTBP3 promotes proliferation and inhibits differentiation of MKN45 cells,^[^ ^33^ ^]^ and interacts with CAV1 to enhance metastasis.^[^ ^34^ ^]^ In addition, PTBP3 plays critical roles in liver cancer, colorectal cancer, pancreatic cancer, and breast cancer.^[^ ^35^, ^36^, ^37^, ^38^ ^]^ These findings underscore the oncogenic potential of PTBP3 and its significant association with poor prognostic outcomes across a range of malignant tumors.

PTBP3, a member of the PTBP family, belongs to the heterogeneous nuclear ribonucleoprotein (hnRNP) family, which includes classical splicing factors typically acting as splicing repressors. The PTBP family consists of three genes: PTBP1, PTBP2, and PTBP3. While extensive research has focused on PTBP proteins, particularly in the context of gastrointestinal tumors,^[^ ^39^ ^]^ their specific role in gastric cancer peritoneal metastasis remains underexplored. In this study, we employed full‐length transcriptome sequencing (third‐generation sequencing) to investigate how PTBP3 regulates the alternative splicing of downstream genes and its involvement in key oncogenic signaling pathways. Our results reveal that a significant number of genes undergo exon skipping events, with these pathways closely related to metabolic processes. Notably, we identified potential associations with genes such as COX11, suggesting that PTBP3 regulates alternative splicing of downstream genes and contributes to the metastatic progression of tumors.

COX11, localized in the mitochondrial inner membrane, plays a critical role in copper ion transport, maintaining copper homeostasis within mitochondria. Cuproptosis, a recently identified mechanism of cellular demise, is triggered by the accumulation of copper ions in the mitochondria when exogenous copper ionophores, such as Elesclomol, are introduced. Catalyzed by FDX1, these ions are converted into toxic monovalent ions, initiating a cascade of biochemical reactions.^[^ ^21^ ^]^ This process leads to reactive oxygen species (ROS) production, depletion of deoxynucleotides, and inhibition of DNA synthesis, ultimately resulting in cell cycle arrest. Furthermore, it activates the p38 signaling pathway while inhibiting the NF‐kB pathway, driving cancer cell death. Excessive copper ion accumulation also causes abnormal aggregation and oligomerization of lipoylated proteins, disrupting iron‐sulfur cluster proteins associated with mitochondrial respiration.^[^ ^40^ ^]^ This disruption triggers a protein toxicity stress response that culminates in cell death. The copper‐induced cell death process is intricately linked to the function of the p53 gene. As a key metabolic regulator, p53 facilitates the transition from glycolysis to mitochondrial metabolism, enhances iron‐sulfur (Fe‐S) cluster biosynthesis, and regulates the levels of the copper chelator GSH.^[^ ^41^ ^]^ Elesclomol, an exogenous copper ionophore, was discontinued in Phase III clinical trials for melanoma treatment; however, it has shown effectiveness in patients with lower plasma lactate dehydrogenase levels. This suggests that Elesclomol may hold therapeutic potential for tumors that are highly sensitive to mitochondrial respiration.^[^ ^42^ ^]^

Our findings indicate that PTBP3 regulates the alternative splicing of COX11, resulting in an increased abundance of short transcripts in metastatic lesions, primarily due to the skipping of exon 4. However, the specific binding sites involved in this process warrant further investigation. Notably, this shorter transcript is correlated with poor patient prognosis and is highly expressed in metastatic tumors. COX11 has two common transcript variants, which may mediate the expression of distinct protein isoforms. The relationship between the structure and function of these proteins requires further exploration. We hypothesize that under normal conditions, COX11 facilitates the transport of copper ions within the mitochondria. However, when an alternative splicing event produces the shorter transcript, the copper transport function may be compromised, leading to a reduction in mitochondrial copper levels. This decrease could hinder the induction of cuproptosis, thus promoting peritoneal metastasis in gastric cancer.

Recent studies have shown that PTBP3 functions as a downstream target of non‐coding RNAs. For example, in liver cancer cell lines, PTBP3 has been identified as a direct target of miR‐297. Overexpression of miR‐297 inhibits the proliferation, migration, and invasion of liver cancer cells by downregulating PTBP3 expression, subsequently suppressing the PI3K/AKT signaling pathway.^[^ ^43^ ^]^ Given that splicing often occurs concurrently with transcription, the elongation rate of the key enzyme RNA polymerase II (RNA pol II) can influence the rate of alternative splicing. Therefore, it is essential to investigate whether transcription factors are involved in the alternative splicing of PTBP3 and to identify the genes regulating PTBP3 expression, which may contribute to its elevated levels in gastric cancer peritoneal metastatic tissues.

Gene therapy is emerging as a promising approach in cancer treatment, with significant advancements in small nucleic acid drug research. Among these, ASO drugs have received the most approvals, primarily targeting genetic disorders. For instance, ASOs have been used to treat conditions such as Duchenne muscular dystrophy, lipoprotein lipase deficiency, and spinal muscular atrophy (SMA). A notable example is Spinraza, approved for SMA treatment, which modulates SMN2 gene splicing to enhance the production of full‐length SMN2 protein, thereby improving motor function in patients. In the context of gastric cancer peritoneal metastasis, ASO drug development often involves chemical modifications to improve efficacy.^[^ ^44^ ^]^ For example, ASOs targeting the SYT13 gene have been modified using amido‐bridged nucleic acid (AmNA) technology to enhance their anti‐metastatic potential in gastric cancer.^[^ ^45^ ^]^ In addition, research into mRNA vaccines is underway to identify new therapeutic targets for this condition. In our study, we designed and synthesized 2′‐methoxyethyl‐modified ASOs targeting the S‐COX11 transcript. These ASOs leverage base‐pairing principles to facilitate the degradation of short transcripts, leading to altered mitochondrial copper ion concentrations and inhibition of gastric cancer peritoneal metastasis. Furthermore, ASOs can be engineered to modify pre‐mRNA splicing patterns. The double‐stranded regions formed by ASO and pre‐mRNA pairing can prevent recognition at specific sites, resulting in the complete excision of complementary exons during pre‐mRNA maturation. This strategy not only modifies the transcripts of target genes but also advances our understanding of mRNA alternative splicing mechanisms. Our findings demonstrate that ASO‐mediated degradation of S‐COX11, in combination with the cuproptosis mechanism, constitutes a potent therapeutic strategy for tumors.

In conclusion, our research identifies PTBP3 as an oncogenic splicing factor and a prognostic marker for gastric cancer peritoneal metastasis. Mechanistically, we found that PTBP3 overexpression promotes alternative splicing of COX11, leading to the increased production of shorter transcripts in peritoneal metastatic tissues. Targeting this short transcript with ASOs, in combination with exogenous copper ionophores, significantly increases intracellular copper concentrations and promotes cuproptosis in gastric cancer peritoneal metastatic cells. Overall, this combined pharmacological approach presents a promising therapeutic strategy for treating GCPM.

## 4 Conclusion

In conclusion, our study identifies PTBP3 as a key regulator of COX11 alternative splicing and highlights its role in the accumulation of shorter transcripts in metastatic cancer lesions. This discovery opens new avenues for therapeutic strategies targeting alternative splicing to modulate copper homeostasis and induce cuproptosis in cancer cells. While the precise mechanisms remain to be fully elucidated, these findings lay the groundwork for future investigations into splicing factor‐targeted therapies in oncology.

## 5 Experimental Section

### Tissue Specimens

The specimens used in this study were obtained from patients admitted to the First Affiliated Hospital of Soochow University for medical treatment. This collection included 102 matched pairs of primary gastric tumors and adjacent normal tissues, sourced from tissue microarrays. In addition, 20 samples of peritoneal metastatic tissues were used for immunohistochemical analysis, organoid extraction, and construction of animal models. The study adhered strictly to the ethical principles outlined in the Declaration of Helsinki and received approval from the Ethics Committee of the First Affiliated Hospital of Soochow University (approval number: 2022164).

### Cell Lines and Cell Culture

Human GC cell lines AGS (RRID: CVCL_0139), HGC‐27 (RRID: CVCL_1297), NCI‐N87 (RRID: CVCL_1603), and MKN45 (RRID: CVCL_0434) were purchased from Procell Life Science & Technology Corporation (Wuhan, China); SNU‐16 (RRID: CVCL_0076) was purchased from Beyotime Biotechnology (Shanghai, China). Human GC cell lines KATOIII (RRID: CVCL_0371), SNU‐1 (RRID: CVCL_0099), and the normal human gastric mucosal epithelial cell line GES‐1 (RRID: CVCL_EQ22) were purchased from Shanghai FuHeng Biotechnology. All cell lines were confirmed to be free from mycoplasma contamination. Cells were cultured in RPMI 1640 or DMEM medium, supplemented with 10% fetal bovine serum (FBS) (FuHeng, Shanghai, China) and 1% Penicillin/Streptomycin (HyClone, USA), in a humidified atmosphere containing 5% CO_2_/95% air at 37 °C.

### RNA Isolation and qRT‐PCR Analysis

Total RNA was isolated from GC tissues or cultured cells using the standard Trizol (Invitrogen) protocol. The integrity, quantity, and purity of the RNA were assessed using a NanoDrop 2000c Spectrophotometer (Thermo Scientific, Wilmington, USA). Briefly, 1 µg of total RNA was reverse transcribed using All‐In‐One 5×RT MasterMix (ABM, Canada). Real‐time quantitative PCR was then performed on an ABI ViiA7 Sequence Detection System (Life Technologies, USA) using SYBR Green Master Mix (ABI). Relative gene expression levels were analyzed using the comparative CT method, where CT represents the cycle threshold number, normalized to GAPDH. The primers used are shown in Table S2 (Supporting Information).

### Western Blot

Cell lysates were prepared in radioimmunoprecipitation assay (RIPA) buffer supplemented with phenylmethanesulfonyl fluoride (PMSF), at a ratio of 100:1 (RIPA:PMSF). Protein concentrations were quantified using the BCA Protein Assay Kit (Epizyme Biotechnology, Shanghai, China). Proteins (10–20 µg) were separated by SDS‐PAGE and electroblotted onto PVDF membranes. Membranes were blocked in TBST containing 5% BSA for 1 h and then incubated with primary antibody at 4 °C overnight. After washing three times with TBST (10 min per wash), membranes were incubated with secondary antibodies diluted in blocking buffer for 1 h at room temperature. Following three additional washes with TBST, enhanced chemiluminescence (ECL) detection was performed using an ECL kit (Shandong Sparkjade Biotechnology Corporation). Densitometric analysis of each band was carried out using ImageJ software for quantification. The following antibodies were used: anti‐PTBP3 (rabbit monoclonal, Cat# 14027‐1‐AP, 1:1000 dilution), purchased from Proteintech (Wuhan, China); anti‐GAPDH (mouse monoclonal, Cat# AF0006, 1:1000 dilution), purchased from Beyotime Biotechnology Company (Shanghai, China); anti‐DLAT (mouse monoclonal, Cat# 68303‐1‐Ig, 1:5000 dilution), purchased from Proteintech (Wuhan, China); anti‐lipoic acid (rabbit polyclonal, Cat# ab58724, 1:1000 dilution), purchased from Abcam (Cambridge, UK); HRP‐conjugated goat anti‐mouse or rabbit antibodies (1:2000 dilution), purchased from Beyotime Biotechnology Company (Shanghai, China).

### 3D Tumor Cells Matrigel Spheroid Formation Assay

Healthy gastric cancer cells were detached from culture dishes (Biosorfa, Zhejiang, China) using trypsin (HyClone, USA) and washed with Dulbecco's phosphate‐buffered saline (DPBS). The cells were counted, suspended in Matrigel (Corning, USA), and seeded into 24‐well plates. Once the Matrigel solidified, DMEM/F12 complete culture medium was added to promote cell differentiation and growth. The complete culture medium contained bFGF (Proteintech, Wuhan, China), EGF (Proteintech, Wuhan, China), BSA (Sigma‐Aldrich, USA), B‐27 (Gibco, Thermo Fisher Scientific, China), and penicillin/streptomycin to stimulate cell differentiation and growth. The ability of the cells to form spheroids was observed under a fluorescence microscope, and the sizes of the spheroids were statistically analyzed.

### EdU Assay

Cell proliferative capacity was assessed using the EdU kit (Beyotime, Shanghai, China). Cells were labeled with EdU reaction solution according to the manufacturer's instructions and incubated at 37 °C for 1–2 h. The cells were then fixed, incubated at room temperature, and protected from light by the addition of Click solution. The nuclei were stained with Hoechst33342 dye (Beyotime, Shanghai, China). Finally, the cells were visualized under a fluorescence microscope (OLYMPUS Corporation, Japan).

### Organoid Construction and Viability Assays

Clinically obtained tumor samples were placed in primary tumor tissue preservation solution (BioGenous, Suzhou, China, Cat: K601005) and washed with DPBS, which was further cleaned by adding 1% penicillin/streptomycin to DPBS. Tumor tissue was trimmed using sterilized scissors and forceps, and the parenchymal portion of the tumor was selected for digestion in primary tumor tissue digestion solution (BioGenous, Suzhou, China, Cat: E238001). Digestion was terminated by adding fetal bovine serum, and the tissue precipitate was obtained by centrifugation. The precipitate was treated with erythrocyte lysate (BioGenous, Suzhou, China, Cat: E238010) and washed again with DPBS. The resulting cell precipitates were resuspended in matrix gel (Mogengel Bio, Xiamen, China, Cat: 082755) and added to 24‐well plates (Sangon Biotech, Shanghai, China), followed by culture in gastric cancer organoid complete medium (Mogengel Bio, Xiamen, China, Cat: MA‐0807T008LP). Well‐grown organoids were detected using an organoid viability assay kit (BioGenous, Suzhou, China, Cat: E238004). The clinical information of the patients from whom the organoids were derived is presented in Table S3 (Supporting Information). After confirming the infection efficiency of the organoids with lentivirus, the effects on organoid viability were compared. On day 7 post‐plating, organoid viability in different groups was assessed using a viability assay kit.

### CRISPR/Cas9

Human‐derived PTBP3 gene sgRNA plasmids were constructed by Hangzhou Guannan Biotechnology Corporation. The pLentiCRISPRv2 vector, containing a puromycin resistance gene, was used. SNU‐1 cells were transfected with plasmids containing single guide RNAs (sgRNAs) and Cas9, targeting the left or right side of the region to be deleted. Colonies were derived from single cells and tested for the deletion of the target region. The knockdown efficiency was assessed by WB assay. The control group was transfected with the sgRNA vector using Lipofectamine 3000 (Invitrogen, Carlsbad, USA) according to the manufacturer's instructions. The sequences of sgRNAs are listed as follows: SgRNA1:GAAGCAGAGATCATATCATT,SgRNA2:GTAATAATTCACCATAGTAA,SgRNA3:GTTACAGATCTTATAACAGT.

### Plasmid and Lentivirus Construction

Lipofectamine 3000 (Invitrogen, Carlsbad, USA) was used as the transfection reagent, and the procedure was performed according to the transfection instructions. Monoclonal cells were selected by adding puromycin after transfection, and knockdown efficiency was evaluated by agarose gel electrophoresis or WB assay. The lentivirus for PTBP3 gene knockdown was constructed using the pLKO.1‐U6‐EGFP‐puro vector, while the overexpression lentivirus was constructed using pCDH‐CMV‐MCS‐EF1‐CopGFP‐T2A‐Puro. A fluorescence microscope was used to capture fluorescence, confirming successful transfection. RT‐qPCR or WB was used to verify transfection efficiency, and puromycin was used to select stably transfected cells. The sequences of knockdown FDX1 are as follows: SiFDX1‐1:GUGAUUCUCUGCUAGAUGUTT(sense), ACAUCUAGCAGAGAAUCACTT(antisense); SiFDX1‐2:CUAACAGACAGAUCACGGUTT(sense), ACCGUGAUCUGUCUGUUAGTT(antisense).

### Application of ASO Drugs

ASOs were designed to target the mRNA of COX11 using standard bioinformatic tools. Sequences were synthesized via solid‐phase synthesis with 2′‐position modifications (MOE, 2′‐O‐methyl) to enhance stability and binding affinity. The purity and structure of the ASOs were confirmed using HPLC and mass spectrometry. ASOs were transfected using Lipofectamine RNAiMAX reagent (Invitrogen, CA, USA) following the manufacturer's protocol. A final concentration of 100 × 10^−9^ m ASO was used for transfection. After 48 h of ASO treatment, organoids were harvested for RNA extraction using TRIzol reagent (Invitrogen, CA, USA). RT‐qPCR was performed to quantify the expression levels of COX11 and other related genes. Nude mice (4–6 weeks old) were injected subcutaneously with 5 × 10^6^ tumor cells. MKN45 cells were injected into the peritoneal cavity of nude mice, and ASO treatment was initiated on day 4 post‐injection. NOD‐SCID immunodeficient mice, aged 5–6 weeks and weighing 16–18 grams, were selected. ASO drugs were administered via intratumoral injection at a dose of 5 nmol, with injections occurring every three days.

### MitoTracker Probes Label Mitochondria

MitoTracker Red CMXRos (Invitrogen, CA, USA), a red fluorescent dye, was used to label mitochondria.^[^ ^46^ ^]^ Initially, cell nuclei were stained with a live cell‐specific dye. Mitochondrial probes were then diluted in basal medium to a working concentration of 100–500 × 10^−9^ m and incubated with cells for 30 min in a controlled environment. After incubation, cells were washed with DPBS, and organoids were pelleted. Specimens were sealed and examined using a confocal microscope, with images captured for documentation.

### Lentivirus Transfection of Organoids

In the container where organoids were to be transfected (e.g., a 96‐well plate or a 24‐well plate), matrix gel was first added and left to solidify, creating a pre‐laid base for subsequent transfection. Organoids were collected from the matrix gel, centrifuged to remove the organoid precipitate, and infected with the lentiviral transfection system. The organoids were resuspended in complete medium containing a specific concentration of viral particles, and a co‐transfection reagent was added to improve infection efficiency. The viral titer was ≈10^8^ TU mL^−1^. The mixture was then inoculated in well plates. After 16 h of infection, the medium containing the viral stock solution and floating organoids was aspirated. A new layer of matrix gel was added over the residual organoids, which solidified, and complete medium was readded for incubation.^[^ ^47^ ^]^ Fluorescence microscopy was used to capture images of the organoids and assess whether fluorescence was present, indicating successful transfection. WB experiments or immunofluorescence assays were performed to verify transfection efficiency.

### Mitochondrial Extraction and Copper Ion Concentration Measurement

Mitochondria were extracted using an intracellular mitochondria extraction kit from Beyotime (Shanghai, China, Cat: C3601) according to the provided instructions. The pellet was gently resuspended in pre‐chilled DPBS and centrifuged at 4 °C for 5 min to pellet the cells. The supernatant was discarded. Next, 1–2.5 mL of mitochondrial isolation reagent was added to resuspend the cells, followed by incubation on ice for 10–15 min. The cell suspension was transferred to a glass homogenizer, homogenized for 10–30 strokes, and homogenization efficiency was assessed. The cell homogenate was then centrifuged at 600 g for 10 min at 4 °C. The supernatant was carefully transferred to a new tube and centrifuged again at 11000 *g* for 10 min at 4 °C. The supernatant was discarded, and the pellet containing isolated mitochondria was resuspended in DPBS. The supernatant was subjected to ultrasonic disruption, and copper content assay reagent (Solarbio, Beijing, China, Cat: BC5750) was added, following the manufacturer's instructions. The absorbance at 580 nm was measured using a multifunctional microplate reader, and the copper concentration was calculated based on the recorded value.

### Agarose Gel Electrophoresis

RNA was first extracted from the samples, and after reverse transcription, cDNA was obtained. Primers and Taq enzyme (Novoprotein, Suzhou, China) were added, and amplification was carried out according to the PCR program. 1 µL of cDNA (at a concentration of 1 µg µL^−1^), 1 µL of each primer (working concentration 10 × 10^−6^ m), and 12.5 µL of 2× Taq Master Mix were combined, and the final volume was adjusted to 25 µL with 9.5 µL of nuclease‐free water. The reverse transcription was performed using the following program: 94 °C for 5 min, followed by 35–40 cycles of 94 °C for 30 s, 55 °C for 30 s, and 72 °C for 45 s, with a final extension at 72 °C for 7 min, and storage at 4 °C indefinitely. Agarose powder was weighed and dissolved in 1× TAE solution, and Gel‐Red dye (Beyotime, Shanghai, China) was added for high‐temperature melting to fully dissolve the agarose powder. To prepare the gel, 1.6 g of agarose powder and 8 µL of Gel‐Red dye were added to 80 mL of 1× TAE solution. The agarose solution was poured into the gel‐making tank, and after solidification, the DNA ladder (Vazyme, Nanjing, China) and prepared samples were added for agarose gel electrophoresis to verify gene transcripts. The electrophoresis parameters were set to 100 V, 100 mA, for 50–60 minutes.

### Immunohistochemistry

5‐µm‐thick paraffin‐embedded sections of clinical specimens, mouse tumor samples, and organoid samples were used for IHC staining following previously reported methods.^[^ ^48^, ^49^ ^]^ Antibodies against PTBP3 (rabbit monoclonal, Cat# 14027‐1‐AP, used at 1:100) were purchased from Proteintech (Wuhan, China). Antibodies against Ki67 (rabbit monoclonal, Cat# GB111499, used at 1:1000) were purchased from Servicebio (Wuhan, China). Antibodies against CK7 (mouse monoclonal, Cat# BD‐PM3054, used at 1:200) were purchased from Biodragon (Suzhou, China). Antibodies against CK20 (rabbit monoclonal, Cat# GB112050‐100, used at 1:1500) were purchased from Servicebio (Wuhan, China). Antibodies against CLDN18.2 (mouse monoclonal, Cat# GB12066‐100, used at 1:500) were purchased from Servicebio (Wuhan, China). Antibodies against DLAT (mouse monoclonal, Cat# 68303‐1‐Ig, used at 1:1000) were purchased from Proteintech (Wuhan, China). Antibodies against Lipoic Acid (rabbit polyclonal, Cat# ab58724, used at 1:1000) were purchased from Abcam (Cambridge, UK). The IHC sections were scanned and analyzed using NanoZoomer S60 (Hamamatsu Photonics) and ImageJ.

### Immunohistochemistry for Organoids

During the procedure, it was observed that the growth of organoids in Matrigel and the difficulty in visually distinguishing smaller spheroids with insufficient diameter required embedding the organoids prior to formal staining. To prepare the embedding matrix, a certain amount of agarose powder was weighed and dissolved in DPBS, adjusting the concentration to 1.5%–3%. The agarose solution was then heated in a microwave until fully dissolved. Using 1.5 mL Eppendorf tubes, the agarose solution was added and allowed to cool slightly. A small eight‐channel tube (commonly used in qPCR experiments) was inserted from the tube's opening just below the surface of the solution, creating a concave indentation in the center of the agarose solution. Once the agarose had nearly solidified, the eight‐channel tube was carefully removed, leaving a cavity in the center, thus forming the mold for embedding the organoids. Organoids were collected, centrifuged to obtain the pellet, and transferred to the bottom of the concave space formed in the mold. The remaining space was sealed with freshly prepared, still‐liquid agarose solution. This process often required repeating the weighing and heating steps twice to ensure proper embedding of the organoid pellet. Afterward, the 1.5 mL Eppendorf tube was cut open from the bottom, and the agarose block containing the organoid was removed and fixed in 4% paraformaldehyde or formalin for further analysis.

### Immunofluorescence of Cells and Organoids

Cells were inoculated in six‐well plates or glass‐bottomed dishes (Biosharp, Beijing, China). When the cell density reached 60%–70%, the medium was removed and the cells were washed with DPBS (Gibco, Thermo Fisher Scientific, China). Then, 4% paraformaldehyde (Biosharp, Beijing, China) was added for fixation, followed by 1% BSA (Beyotime, USA) for permeabilization. After aspiration of the paraformaldehyde, Triton X‐100 (Sigma‐Aldrich, USA) was added for further permeabilization, and 1% BSA (Beyotime, Shanghai, China) was applied for blocking. The cells were washed again and incubated with primary antibodies overnight. This was followed by incubation with secondary antibodies and nuclear staining with Hoechst33342 (Beyotime, Shanghai, China). Finally, the cells were observed and photographed using a confocal scanning microscope (OLYMPUS Corporation, Japan). Organoids were collected in centrifuge tubes (Promethe, Shanghai, China) for experimentation and then dropped onto slides (Citotest, Jiangsu, China) for observation and imaging. The antibodies used were as follows: anti‐PTBP3 (rabbit monoclonal, Cat# 14027‐1‐AP, 1:400 dilution), anti‐TOM20 (mouse monoclonal, Cat# 66777‐1‐Ig, 1:400 dilution), anti‐DLAT (mouse monoclonal, Cat# 68303‐1‐Ig, 1:400 dilution), anti‐Ki67 (rabbit monoclonal, Cat# GB111499, 1:400 dilution), anti‐CK7 (mouse monoclonal, Cat# BD‐PM3054, 1:400 dilution), anti‐CK20 (rabbit monoclonal, Cat# GB112050‐100, 1:400 dilution), anti‐CLDN18.2 (mouse monoclonal, Cat# GB12066‐100, 1:400 dilution), and anti‐ACTN1 (mouse monoclonal, Cat# BD‐PT0101, 1:400 dilution). All antibodies were purchased from Proteintech (Wuhan, China), Servicebio (Wuhan, China), or Biodragon (Suzhou, China).

### Soluble and Insoluble Fraction Isolation

Organoids were cultured in a matrix gel and subsequently harvested for lysis in ice‐cold NP40 lysis buffer containing 20 × 10^−3^ m Tris (pH 7.4), 150 × 10^−3^ m NaCl, and 1% NP40, supplemented with an EDTA‐free protease inhibitor cocktail (B14001, Selleck) for 30 min. After lysis, the cell extracts were centrifuged at 14000 rpm at 4 °C for 30 min. The supernatant and pellet were then separated. The supernatant was mixed with 5× SDS loading buffer, while the pellet was resuspended in 1× SDS loading buffer. Both fractions were boiled and prepared for Western blotting.

### Animal Model Construction

*Establishment of a Peritoneal Injection Model of Gastric Cancer Cells*: Female nude mice aged 5–6 weeks and weighing 16–18 g were used in the study. MKN45 cells, labeled with luciferase, were cultured in an appropriate medium after lentiviral transduction for overexpression and were maintained until reaching the logarithmic growth phase. The cells were then digested with trypsin, collected by centrifugation, and counted before being resuspended in DPBS at a concentration of one million cells for injection. Luciferin potassium salt was injected into the peritoneal cavity to activate the luciferase and generate fluorescence. A small animal in vivo imaging system (KODAK, USA) was used to periodically monitor the fluorescence in the peritoneal cavity, allowing for assessment of tumorigenesis and observation of progression.

*Establishment of a PDOX Model*: NOD‐SCID immunodeficient mice (RRID: IMSR_JAX:001303), aged 5–6 weeks and weighing 16–18 g, were selected. Well‐growing organoids were isolated from the matrix gel, collected by centrifugation, and washed with DPBS. The extracted organoids were injected subcutaneously to establish a subcutaneous tumor model, with the addition of a high‐concentration matrix gel during injection to facilitate tumor formation, using a minimum of one million cells.^[^ ^50^ ^]^ Tumor volume (cm^3^) = 0.5 × Tumor length × Tumor width^2^. All animal studies (including the euthanasia procedure) were conducted in compliance with Soochow University institutional animal care regulations and in accordance with AAALAC and IACUC guidelines (approval numbers: 202403A0196, 202403A0197).

### Full‐Length Transcriptome Sequencing

RNA was extracted from the samples prepared for sequencing. A total of 500 ng of RNA passed quality control and was adjusted to 9 µL using nuclease‐free water. Reverse transcription primers were added for the reverse transcription reaction, followed by amplification. After purification, sequencing adapters were ligated to the magnetic beads of AMPure beads, and the library was loaded onto the R9.4 sequencing chip. Sequencing was performed on the PromethION sequencer (Oxford Nanopore Technologies, Oxford, UK) for 48–72 h. The library was loaded onto the R9.4 microarray, and sequencing was conducted on the PromethION sequencer (Oxford Nanopore Technologies, Oxford, UK) for 48–72 h.^[^ ^51^ ^]^ The sequencing and subsequent analysis were performed by Wuhan Benagene Corporation.

### Bioinformatic Analysis

Single‐cell sequencing analysis was conducted by Beijing Novogene Technology Corporation, which integrated public data, performed cell annotation and definition, and carried out inferCNV analysis. Tumor cells in the samples were isolated, and differentially expressed genes between the two groups were compared. Further functional enrichment analyses, including GO analysis, KEGG analysis, and GSEA analysis, were also performed.

### Kaplan–Meier Survival Analysis

Overall survival curves for the patient cohort were plotted using GraphPad. High and low expression were based on the mRNA levels of PTBP3 in tissue samples. An online Kaplan–Meier plotter database (https://kmplot.com/analysis/) was used to analyze the association between the mRNA expression levels of genes of interest and the survival outcomes of patients with gastric cancer.

### Statistical Analysis

Statistical analyses were performed using SPSS 27.0 software (IBM Corporation) and GraphPad Prism 10. Continuous data are presented as means ± standard deviation (SD), and differences among experimental groups were analyzed using one‐way ANOVA or Student's *t*‐test. Frequencies of categorical variables were compared using Pearson's χ^2^ test. The survival curve was generated using the Kaplan–Meier method and compared by the log‐rank test. A *p*‐value < 0.05 was considered statistically significant. Results from multiple comparison tests were obtained using Python (Python 3.8), utilizing the Benjamini–Hochberg multiple comparison function from the statsmodels package. The results were further validated using the multiple comparison testing tool (www.multipletesting.com) (FDR < 5%).^[^ ^52^, ^53^ ^]^

### Ethics Approval and Consent to Participate

All study participants provided informed consent, and the study design was approved by the Institutional Ethics Committee of the First Affiliated Hospital of Soochow University (approval number: 2022164).

## Conflict of Interest

The authors declare no conflict of interest.

## Author Contributions

Y.Z., C.D., and X.S. contributed equally to this work. Y.Z., D.L., C.D., and X.S. conceived and designed the experiments. Y.Z., D.L., C.D., and X.S. wrote, reviewed, and revised the manuscript; P.W., T.C., and W.L. developed the methodology; X.S. and P.L. collected clinical samples; C.X. analyzed and interpreted the data; K.D., D.L., and J.Z. supervised the study. All authors read and approved the final manuscript.

## Supporting information



Supporting Information

Supporting Information

Supporting Table 3

## Data Availability

The data that support the findings of this study are available from the corresponding author upon reasonable request.
