# Supplementary material for: Targeting PTBP3‐Mediated Alternative Splicing of COX11 Induces Cuproptosis for Inhibiting Gastric Cancer Peritoneal Metastasis
Source: Adv Sci (Weinh). 2025 Apr 24;12(21):2415983. doi: 10.1002/advs.202415983 (PMC12140345; doi:10.1002/advs.202415983)
Supplement: Supplementary file 1 — Supporting Information [file ADVS-12-2415983-s002.docx]

**Supplementary figures**

Figure S1

**
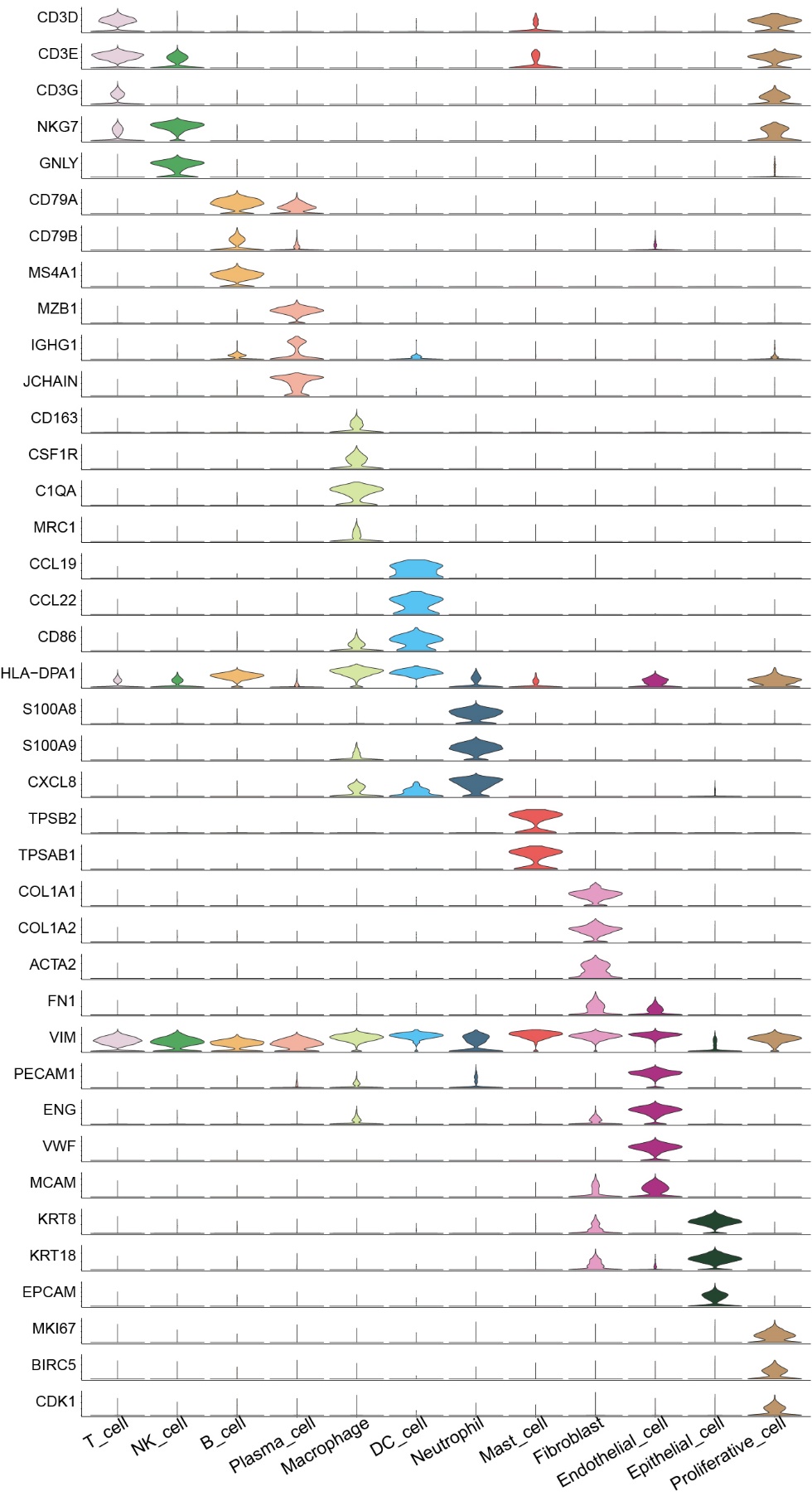
**

**Fig. S1** Specific marker genes used to identify and annotate various cell subpopulations in single-cell sequencing. Figure S2

**
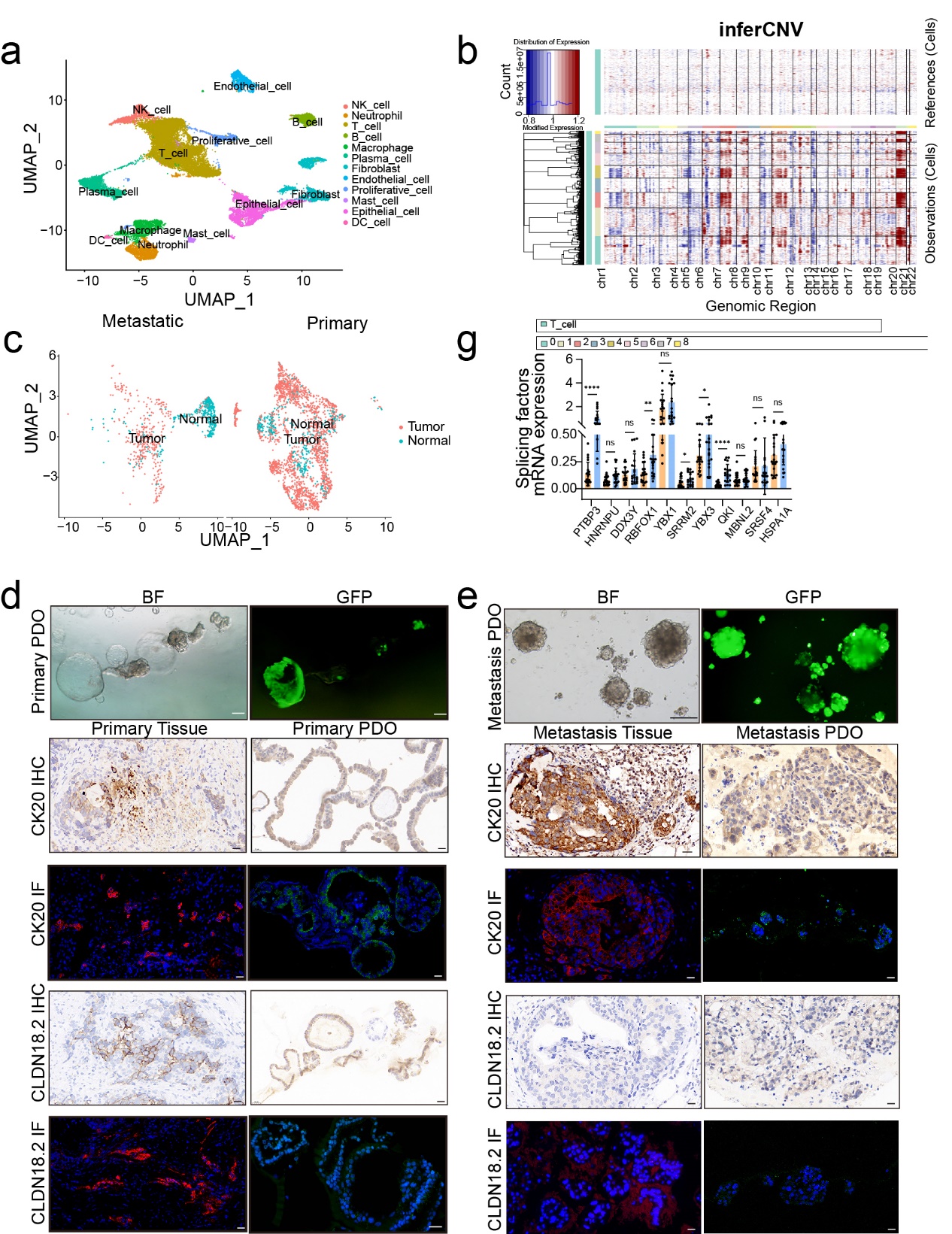
**

**
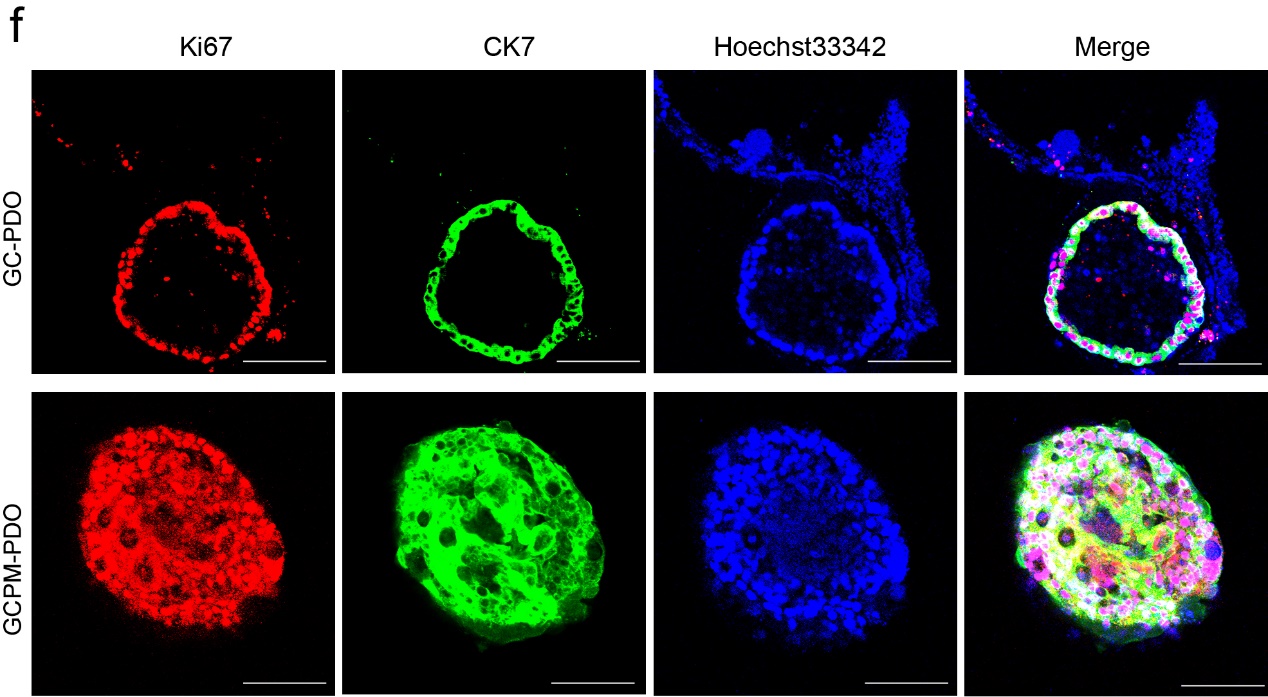
**

**Fig. S2 a.** Uniform Manifold Approximation and Projection (UMAP) plots showing the full sample set included in the analysis, with annotated cell types listed on the right. **b.** InferCNV analysis of epithelial cells was conducted to distinguish tumor cells from normal cells, with T-cells serving as the reference. **c.** Tumor cells and normal cells were isolated from epithelial cells, and differences between the two groups are demonstrated. **d.** Diagrams showing various indicators of primary organoids and their source tissues. The scale for the organoid bright-field and corresponding GFP lentiviral fluorescence image is 200 µm; the scale bar for all other indicators is 20 µm. **e.** Diagrams showing various indicators of metastatic organoids and their source tissues. The scale for the organoid bright-field and corresponding GFP lentiviral fluorescence image is 200 µm; the scale bar for all other indicators is 20 µm. **f.** Ki67 and CK7 fluorescence double staining of organoids derived from primary gastric cancer (upper) and metastatic gastric cancer (lower). Scale bar, 100 µm. **g.** Histogram of mRNA expression levels of common genes in primary and metastatic tissues (data are expressed as mean ± s.d., n = 20). Statistical analysis: Unpaired Student’s t-test; ns, no significant, *P < 0.05, **P < 0.01, ***P < 0.001, ****P < 0.0001.

Figure S3

**
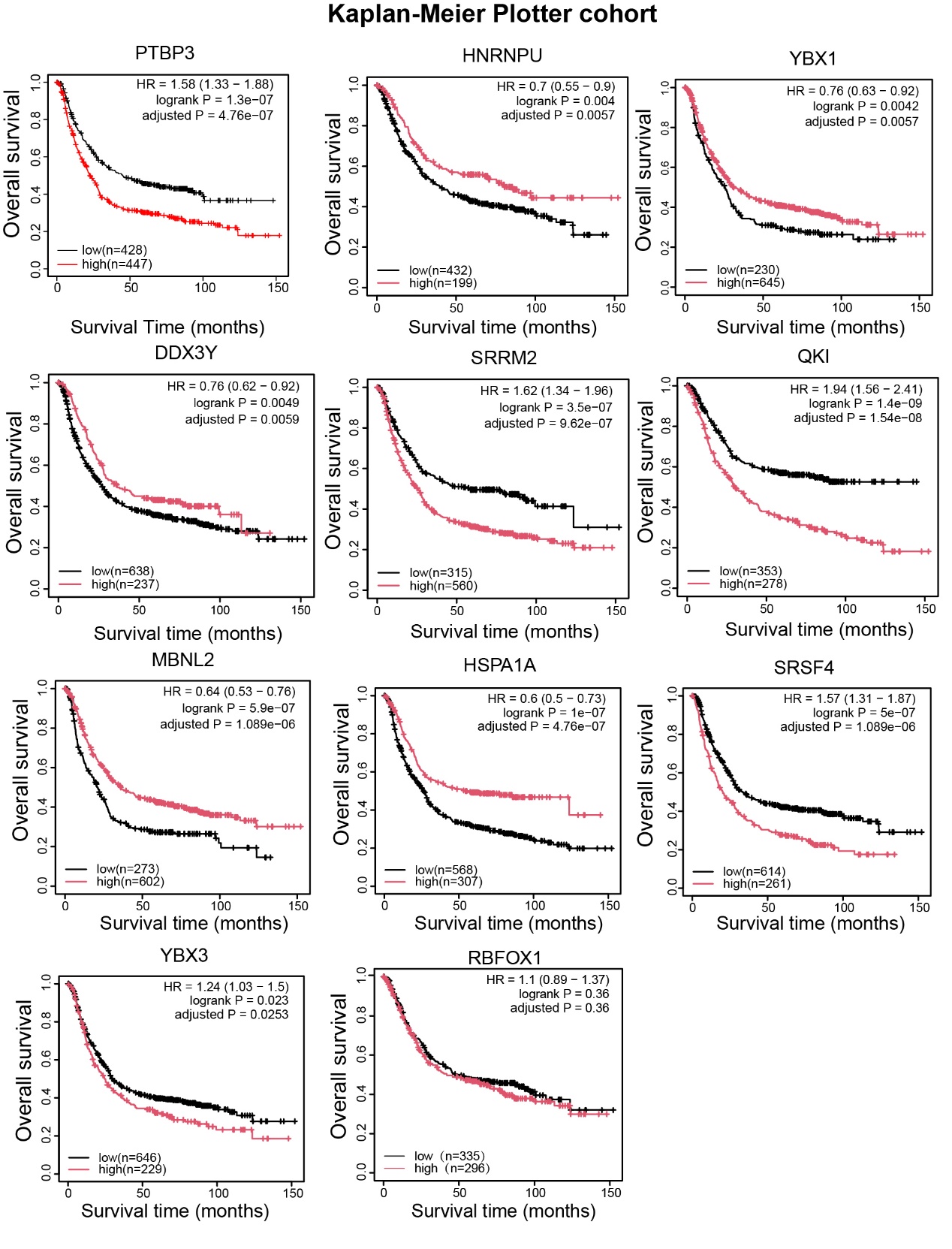
**

**Fig. S3** Kaplan-Meier analysis of the correlation between the expression of listed genes and overall survival of GC patients based on the Kaplan-Meier Plotter cohort. The results of the multiple comparison test were calculated using the Benjamini-Hochberg method (FDR < 5%).Figure S4

**
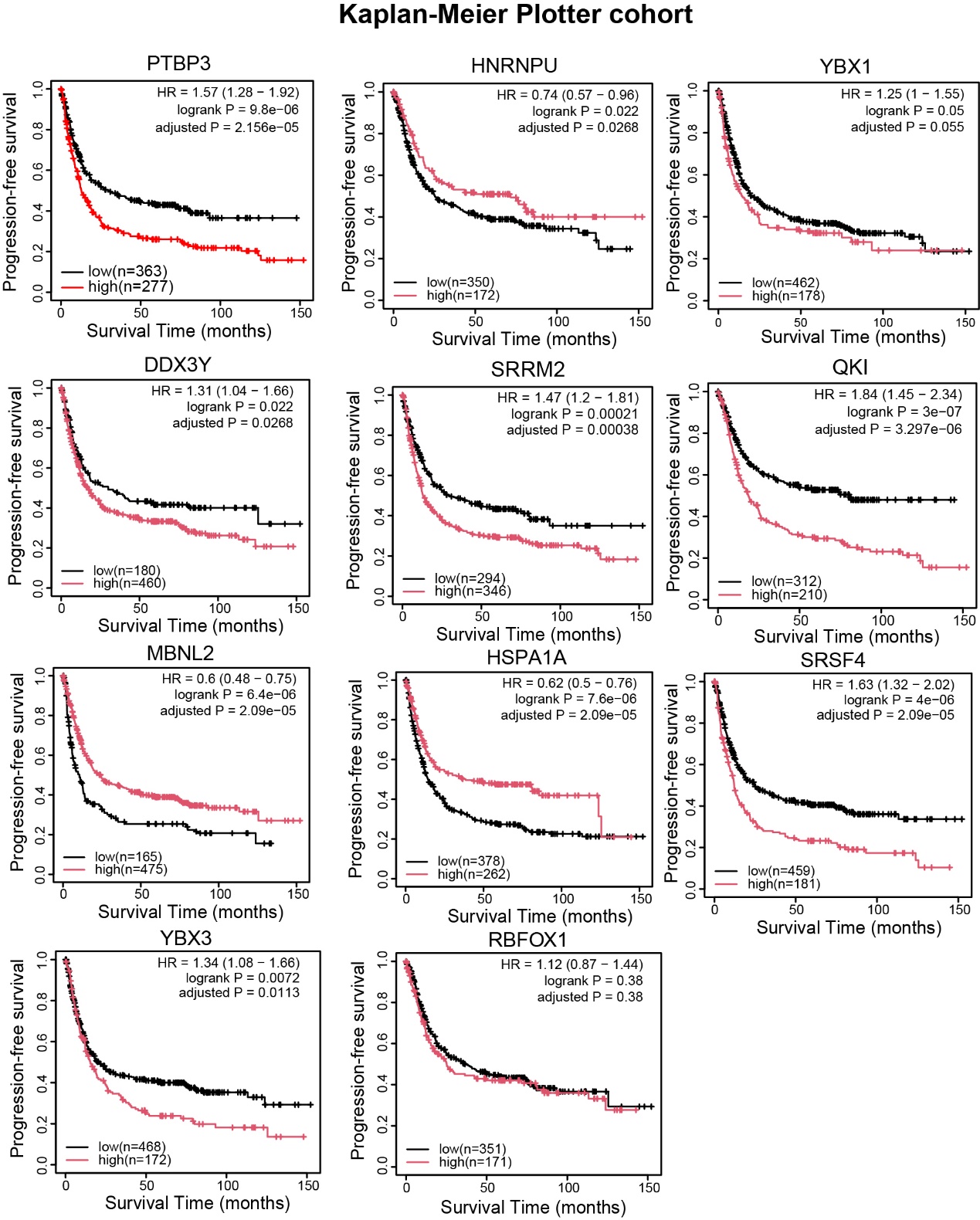
**

**Fig. S4** Kaplan-Meier analysis of the correlation between the expression of listed genes and progression-free survival of GC patients based on the Kaplan-Meier Plotter cohort. The results of the multiple comparison test were calculated using the Benjamini-Hochberg method (FDR < 5%).Figure S5

**
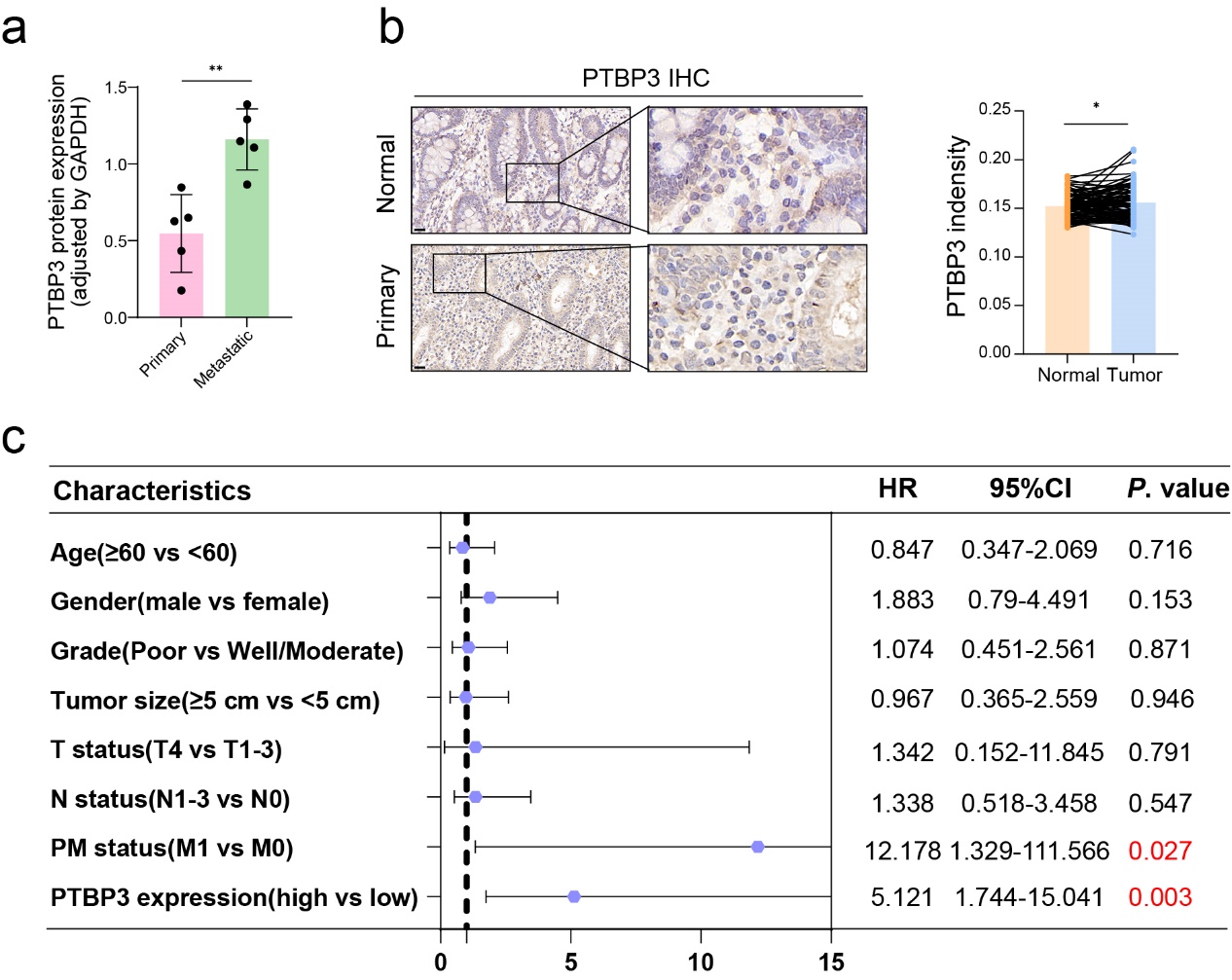
**

**Fig. S5 a.** Quantitative analysis of PTBP3 expression in organoids derived from primary and metastatic gastric cancer tissues. Data are expressed as mean ± s.d., n = 5. Statistical analysis: Unpaired Student’s t-test, **P < 0.01. **b.** Immunoblot analysis comparing gastric cancer tissues to adjacent normal tissues, with bar graphs demonstrating statistically significant differences in PTBP3 expression. Scale bars, 2000 µm. Data are expressed as mean ± s.d., n = 102. Statistical analysis: Unpaired Student’s t-test, *P < 0.05. **c.** Multivariate Cox regression model analysis of the relationship between PTBP3 expression and prognosis of gastric cancer. *P < 0.05, **P < 0.01.

Figure S6

**
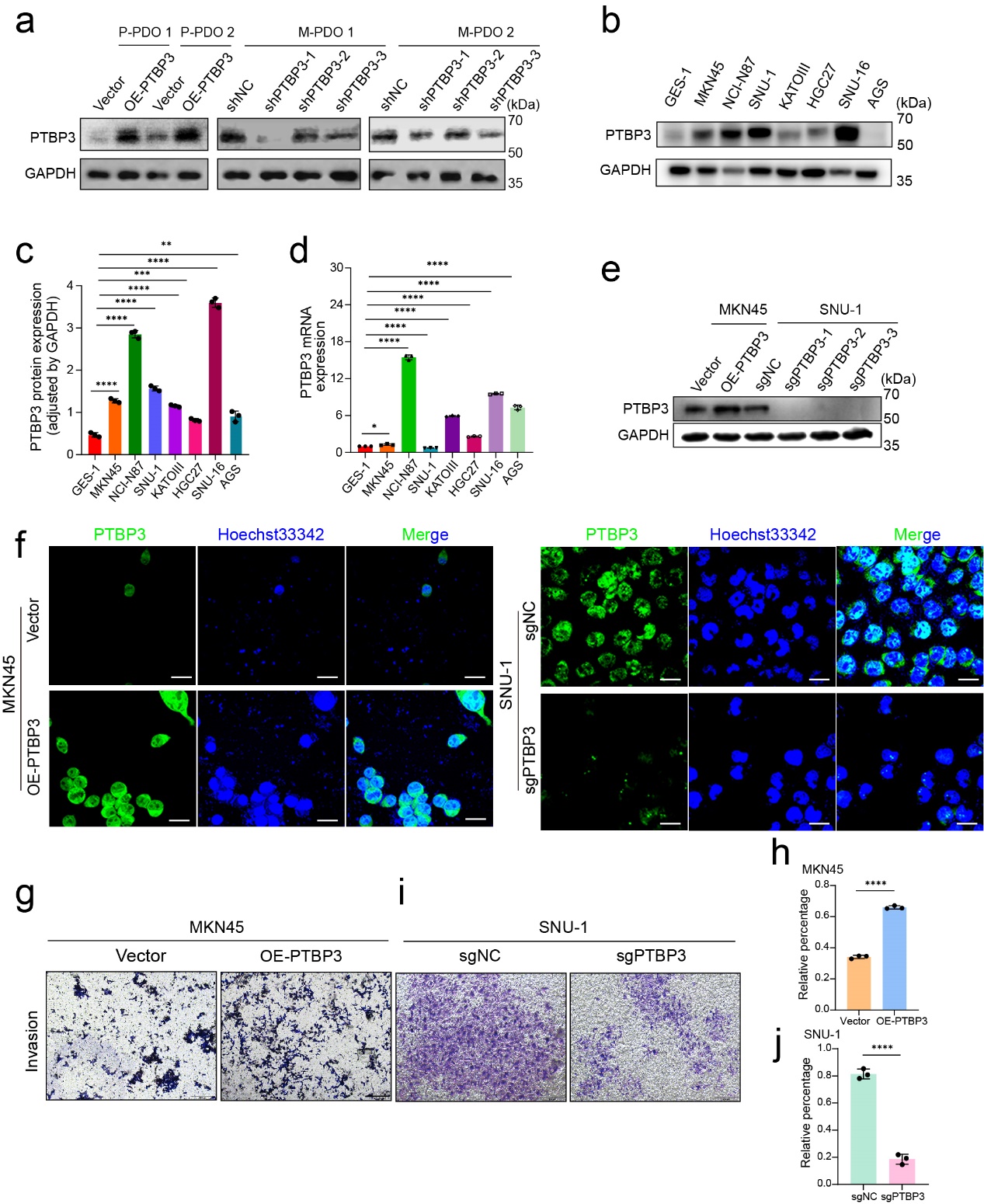
**

**Fig. S6** **a.** Western blot analysis showing PTBP3 overexpression (left) and knockdown (right) in organoids. **b, c.** Western blot assay of PTBP3 expression in different gastric cancer cells, with further semiquantitative analysis based on GAPDH expression. Data are expressed as mean ± s.d., n = 3. Statistical analysis: Unpaired Student’s t-test, **P < 0.01, ***P < 0.001, ****P < 0.0001. **d.** PTBP3 mRNA expression in different gastric cancer cells. Data are expressed as mean ± s.d., n = 3. Statistical analysis: Unpaired Student’s t-test, *P < 0.05, ****P < 0.0001. **e.** Immunoblot analysis of MKN45 cells transduced with empty vector control lentivirus (Vector) or PTBP3 overexpression lentivirus (OE-PTBP3) (left), and SNU-1 cells transduced with CRISPR-Cas9 control plasmids (sgNC), PTBP3 sgRNA-1 (sgPTBP3-1), PTBP3 sgRNA-2 (sgPTBP3-2), and PTBP3 sgRNA-3 (sgPTBP3-3) plasmids (right). **f.** Immunofluorescence assay to detect PTBP3 expression in different cell lines. Left, MKN45 expression shown for overexpression (bottom) and vector control (top) groups; Right, SNU-1 expression for negative control group (sgNC, top) and knockout group (sgPTBP3, bottom). Red: PTBP3, green: CK7, blue: Hoechst33342. Scale bars, 30 µm. **g, h.** Transwell assay of MKN45 cells with cell count statistics. Scale bar: 200 µm. Data are expressed as mean ± s.d., n = 3. Statistical analysis: Student’s t-test, ****P < 0.0001. **i, j.** Transwell assay of SNU-1 cells with cell count statistics. Scale bar: 200 µm. Data are expressed as mean ± s.d., n = 3. Statistical analysis: Unpaired Student’s t-test, ****P < 0.0001.Figure S7

**
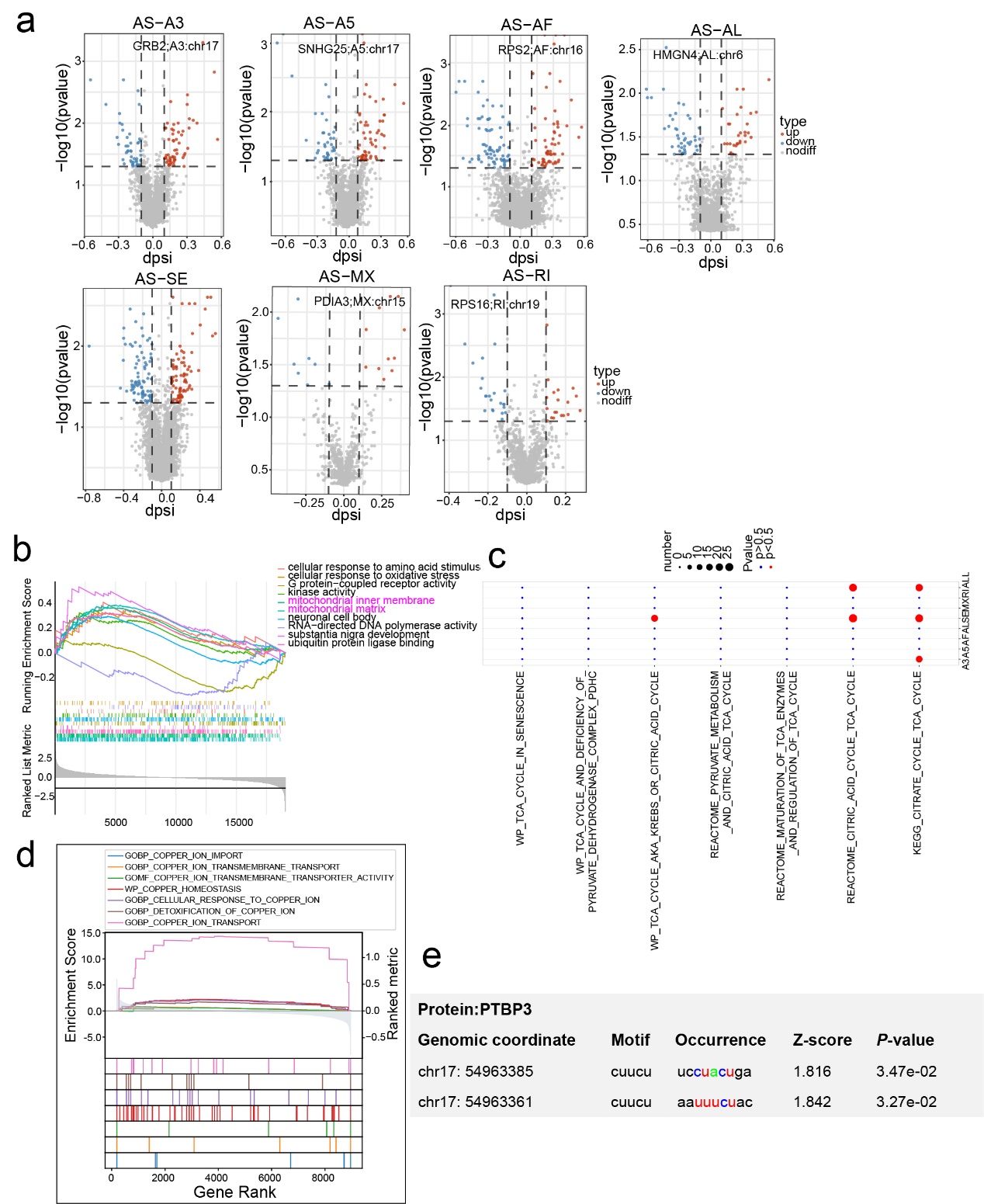
**

**Fig. S7** **a.** Volcano plot showing genes associated with common types of splicing, with the most prominent genes in each type labeled. **b.** Gene Set Enrichment Analysis (GSEA) reveals that differential gene function correlates with processes such as mitochondrial function. **c.** Bubble diagram demonstrating that among the genes undergoing variable splicing, differential gene function is closely linked to the TCA cycle. **d.** Single-cell sequencing data analyzed using GSEA, showing associations with pathways related to copper transport and copper homeostasis. **e.** Prediction of PTBP3 binding sites on COX11 pre-mRNA sequences enriched in C/U pyrimidines, using the RBPmap online tool (http://rbpmap.technion.ac.il).Figure S8

**
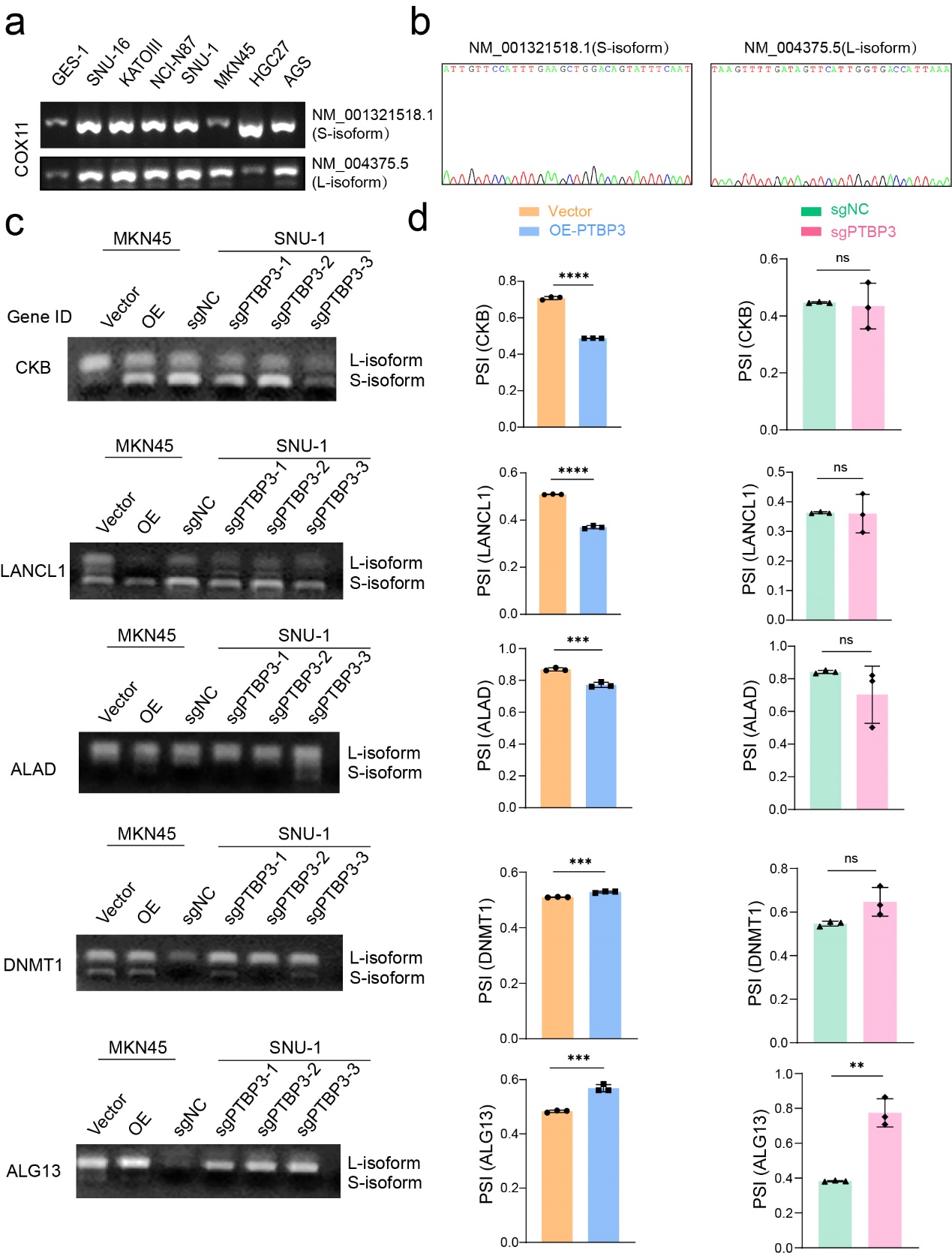
**

**Fig. S8** **a, b.** Agarose gel electrophoresis experiments demonstrating the expression of two major COX11 transcripts in different gastric cancer cell lines, with Sanger-sequenced base sequence diagrams displayed on the right. **c, d.** Agarose gel electrophoresis analysis of selected genes, with statistical analysis of different transcript profiles based on gray values. Statistical tests involved: Unpaired Student’s t-test, ns, no significant, **P < 0.01, ***P < 0.001, ****P < 0.0001, Data are expressed as mean ± s.d., n = 3.Figure S9

**
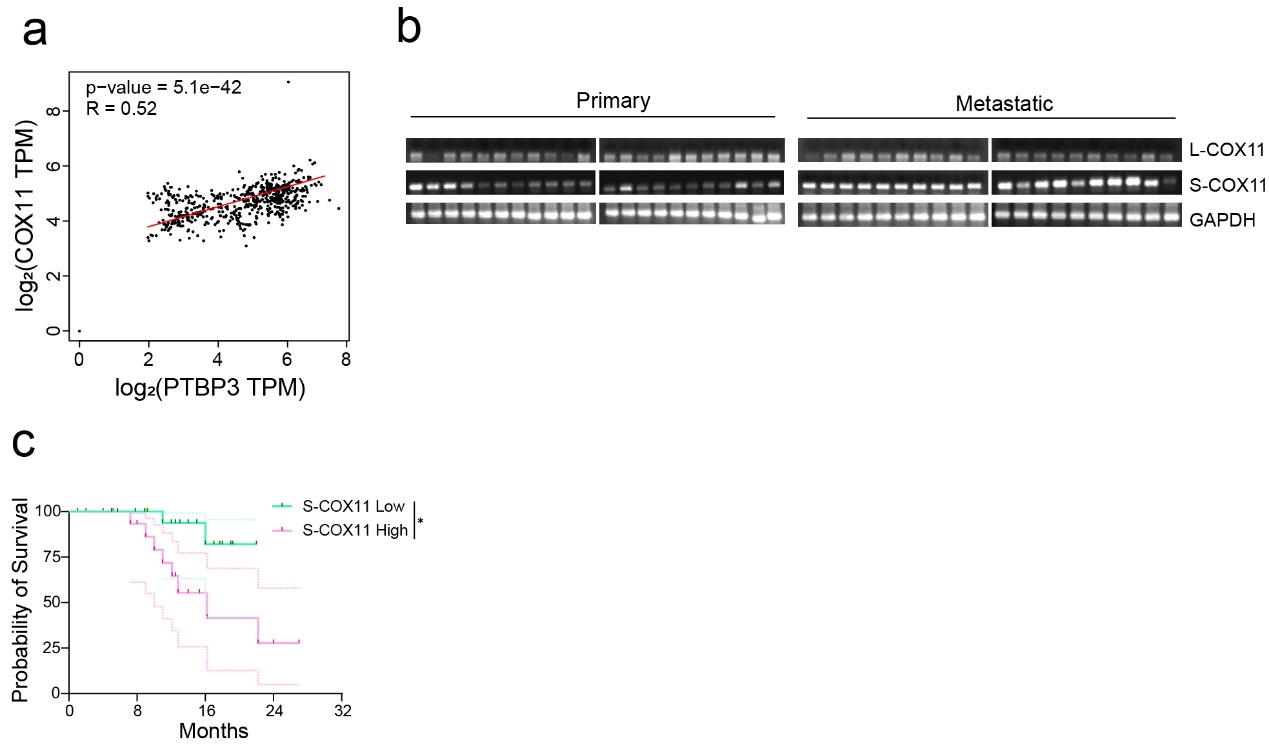
**

**Fig. S9** **a.** Correlation analysis of COX11 TPM and PTBP3 TPM in gastric cancer, showing a positive correlation between the two by Spearman analysis. **b.** Agarose gel electrophoresis experiments on the expression of short and long COX11 transcripts in 22 primary and 20 metastatic gastric cancer tissues. **c.** Survival analysis curves for patients with high and low S-COX11 expression in panel b, plotted by GraphPad.Figure S10

**
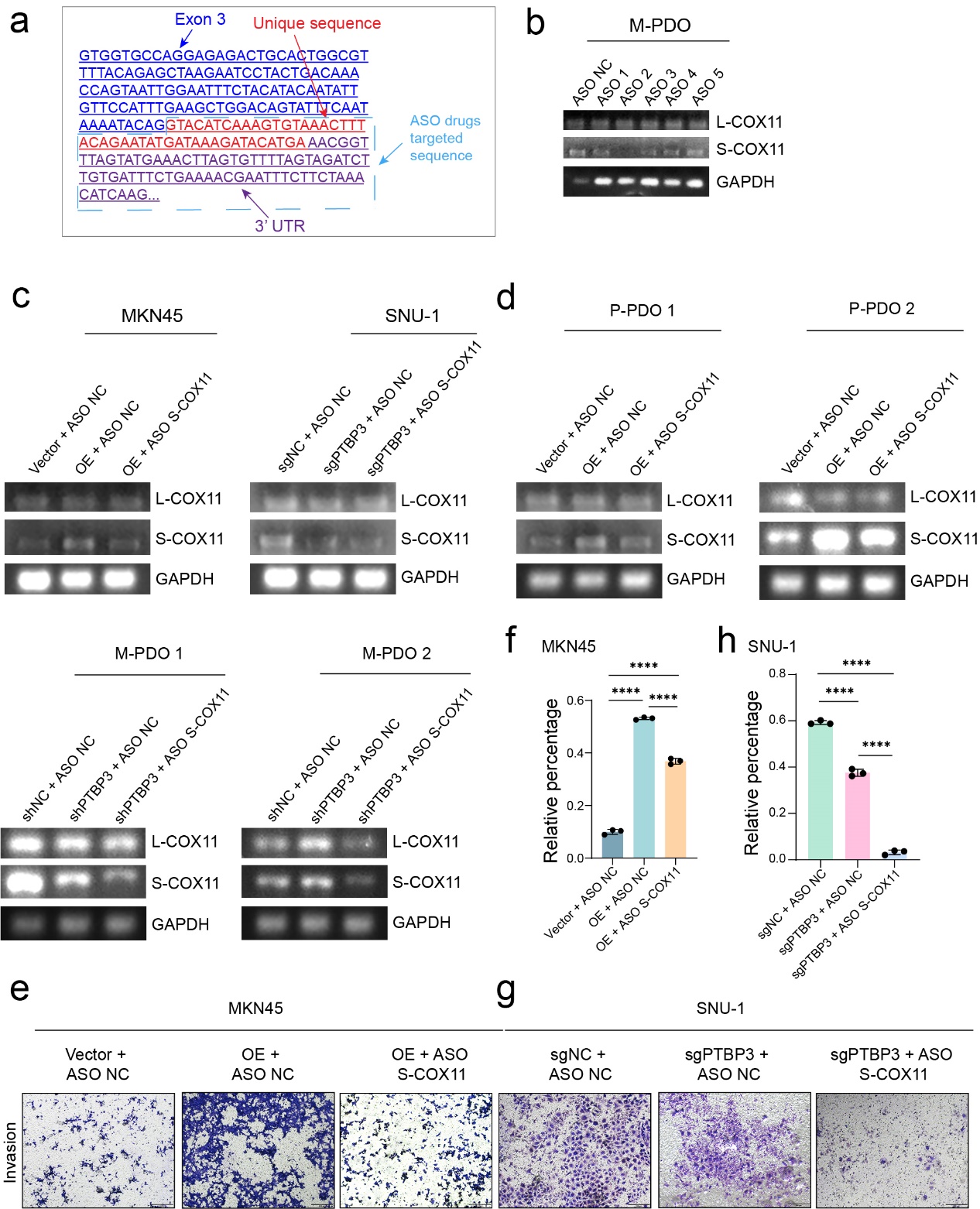
**

**Fig. S10 a.** Schematic representation of the ASO drug's site of action, designed to target the short transcript of COX11. **b.** Agarose gel electrophoresis validating the sequences used in the ASO drug targeting. **c.** Transcript expression comparison between groups in the response experiment. Left: MKN45; Right: SNU-1. **d.** Transcript expression comparison between groups in the response experiment. Top: P-PDO; Bottom: M-PDO. **e, f.** Transwell assay of MKN45 cells with cell count statistics. Scale bar: 200 µm. Statistical tests involved: ****P < 0.0001, Unpaired Student’s t-test; Data are expressed as mean ± s.d., n = 3. **g, h.** Transwell assay of SNU-1 cells with cell count statistics. Scale bar: 200 µm. Statistical tests involved: ****P < 0.0001, Unpaired Student’s t-test; Data are expressed as mean ± s.d., n = 3.Figure S11

**
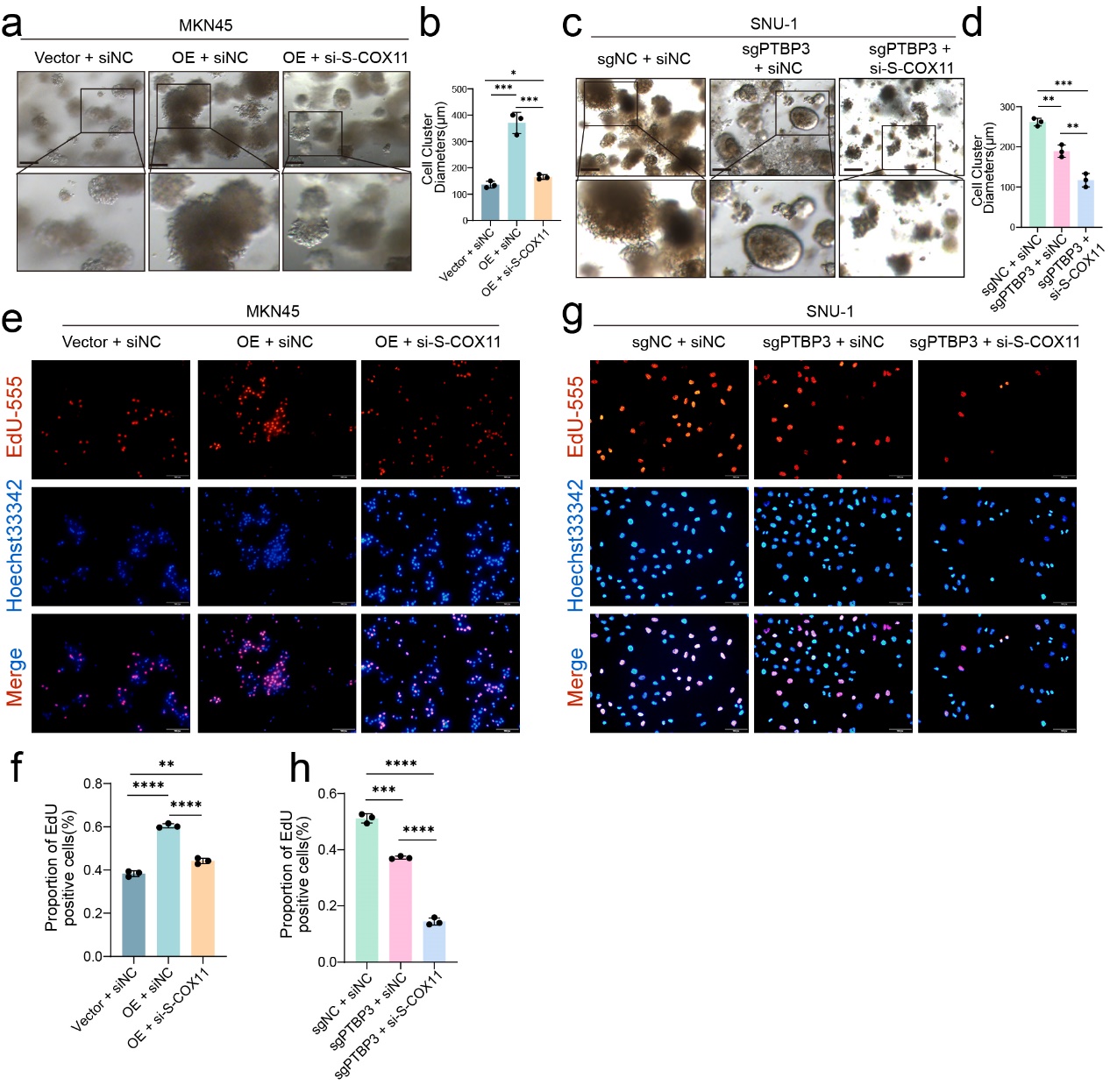
**

**Fig. S11** **a, b.** Representative images (left) and quantitative comparison (right) of 3D Matrigel spheroid formation in si-S-COX11 rescue assays in MKN45 cells. Top: normal field; Bottom: magnified image. Scale bars: 200 µm. **c, d.** Representative images (left) and quantitative comparison (right) of 3D Matrigel spheroid formation in si-S-COX11 rescue assays in SNU-1 cells. Top: normal field; Bottom: magnified image. Scale bars: 200 µm. **e.** Representative images of EdU assay in si-S-COX11 rescue assays in MKN45 cells. Scale bars: 100 µm. **f.** Quantitative comparison of EdU assay in si-S-COX11 rescue assays in MKN45 cells (n = 3 for each group; data are mean ± s.d.). **g.** Representative images of EdU assay in si-S-COX11 rescue assays in SNU-1 cells. Scale bars: 100 µm. **h.** Quantitative comparison of EdU assay in si-S-COX11 rescue assays in SNU-1 cells (n = 3 for each group; data are mean ± s.d.). In all panels, data are expressed as mean ± s.d., n ≥ 3. Unpaired two-tailed Student’s t-test (b, d, f, h). *P < 0.05, **P < 0.01, ***P < 0.001, ***P < 0.0001.Figure S12

**
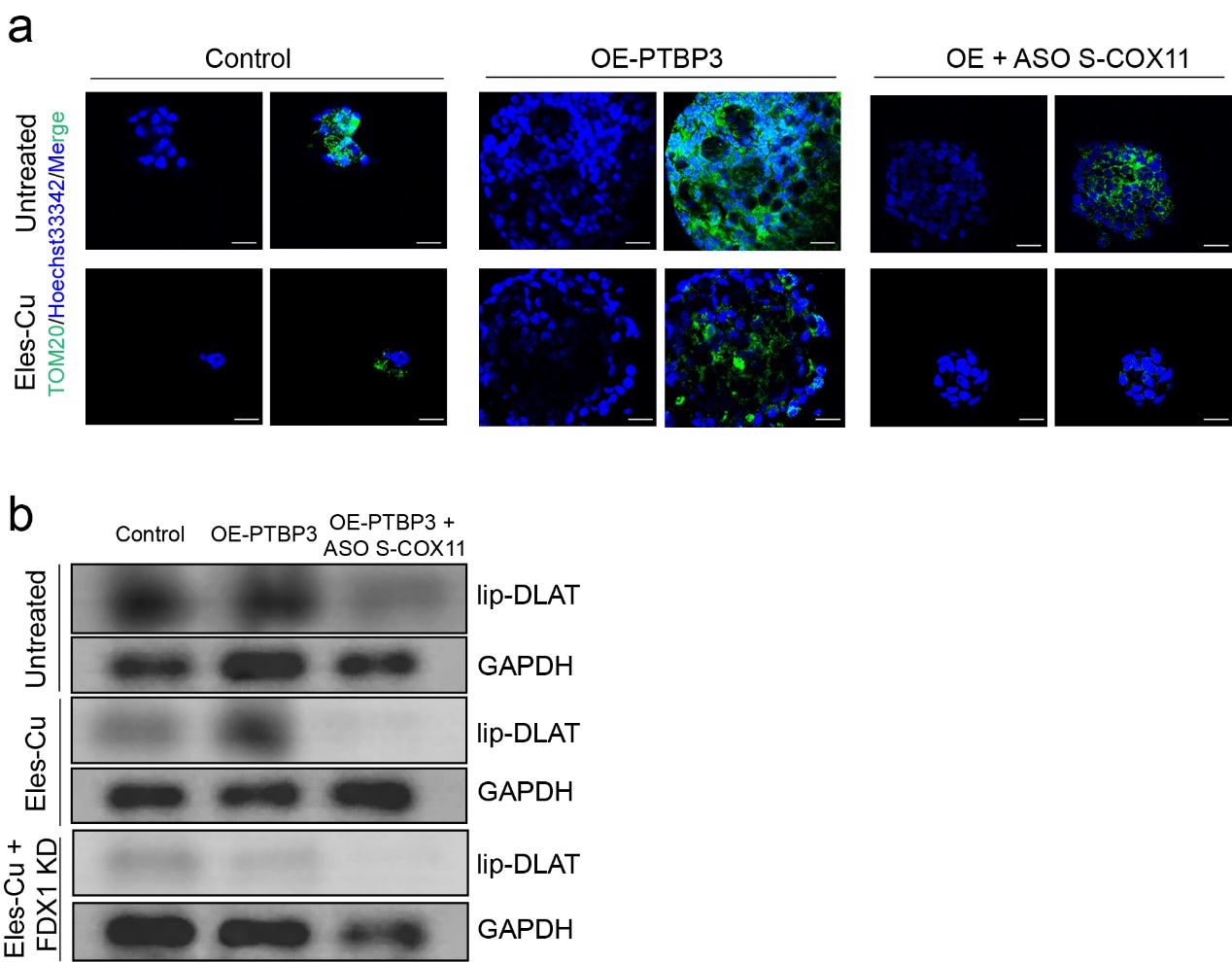
**

**Fig. S12** **a.** TOM20 was analyzed by immunofluorescence (IF). Green: TOM20; Blue: Hoechst33342. Upper: untreated; Lower: treated with Eles-Cu combination at a drug concentration of 10^-8^ M for 3 days. Scale bars: 30 µm. **b.** Western blot analysis to validate the expression of lipoylated DLAT protein in different samples after 24 hours.Figure S13

**
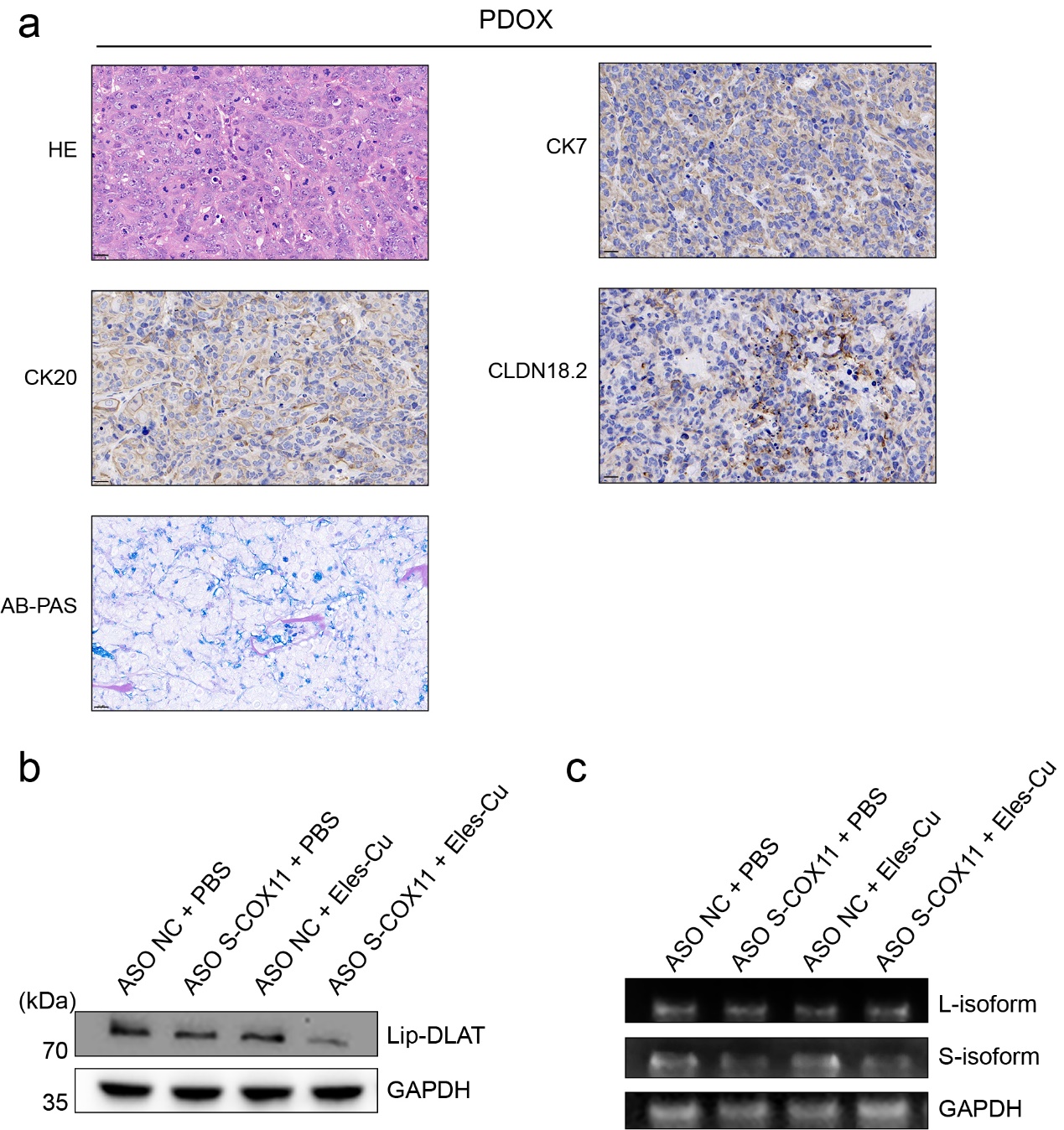
**

**Fig. S13** **a.** Representative images of HE, CK7 IHC staining, CK20 IHC staining, CLDN18.2 IHC staining, and AB-PAS in GCPM PDOX P0 tumors. Scale bars: 20 µm. **b.** Western blot analysis to detect the levels of lipoylated DLAT in the samples. **c.** Agarose detection of long and short COX11 transcripts in four groups.

**Table S1**

**
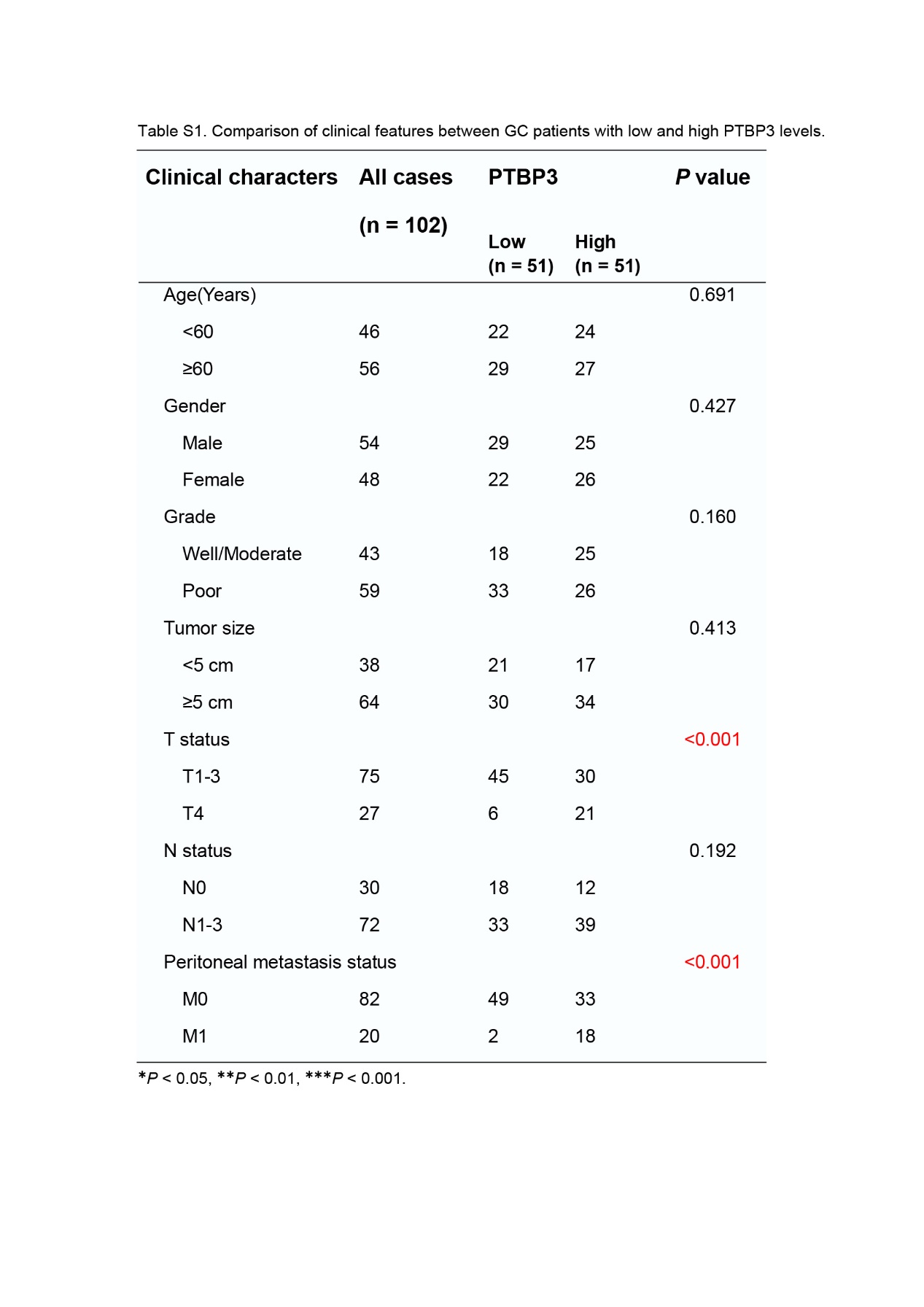
**

**Table S2** **Primers for RT-PCR and RT-qPCR**

| PTBP3 | FP: CTTCCTCTGCTCGCGGTTAG  RP: CTCATCAGATCCCCGCCATC |
| --- | --- |
| GAPDH | FP: GACAGTCAGCCGCATCTTCT  RP: GCGCCCAATACGACCAAATC |
| COX11  (Short isoform) | FP: ATGTGGTGCCAGGAGAGACT  RP: AGCAGATATCCATTTGGTTTGGTT |
| COX11  (Long isoform) | FP: ACTGGGAAGCTGGAGAGTCA  RP: AACCGGTACCAGTAGACCCA |
| RBFOX1 | FP: GGATTGAGAGTCCTTGCGCT  RP: GTTTCCTAGAACTCCCGGCG |
| DDX3Y | FP: AACACCACTACAAGGGTGGC  RP: CGACTAGCACCAAAGCCAGA |
| HNRNPU | FP: GTGCTCGCTCGCGCC  RP: AACAGGCGAGGAACTCATGG |
| YBX1 | FP: GCCGGCTTACCATCTCTACC  RP: GGTCAACGGGCAAAAAGCAA |
| SRRM2 | FP: GGACAAAAAGCGAAAGCGGT  RP: TTAAGGGGGTGGTTGCAAGG |
| QKI | FP: CCCAACTTCTGCGGGATCTT  RP: TGCAAACTTGTCTTGCCTCTC |
| MBNL2 | FP: CTGTGTCTGAGGAGCCTTGG  RP: TGCTGACGCTCACAGCTATT |
| HSPA1A | FP: AGCTGGAGCAGGTGTGTAAC  RP: CAGCAATCTTGGAAAGGCCC |
| SRSF4 | FP: GTGGAGCGCTTCTTTAAGGG  RP: TTCGGCTTCTGCTCTTACGG |
| YBX3 | FP: TACCGTAGCAGGGGACCTC  RP: AGCCTGGTGTTACTCAGCAC |
| LANCL1 | FP: AGGAAGAGAAACTTCACGGCA  RP: ACTTCATGTCCTGTGTGAGGT |
| ALAD | FP: ACGCGGTCTGTGGGAGA  RP: TGAGGTTGGAGGCATTGAGG |
| ALG13 | FP: GCCATGAAGTGCGTGTTTGT  RP: AAGAGGCATCCTTGGCAGTC |
| DNMT1 | FP: TATCCGAGGAGGGCTACCTG  RP: ATGAGCACCGTTCTCCAAGG |
| CKB | FP: GTACATCATGACCGTGGGCT  RP: ACCGCGAGCTTCTCGATG |
